# StACS3-mediated drought stress adaptation in potato involves interactions with StPP2C2 and St14-3-3 proteins

**DOI:** 10.3389/fpls.2025.1671817

**Published:** 2025-10-30

**Authors:** Sadia Hamera, Safee Ullah Chaudhary, Heiko Lemcke, Martin Sklorz, Florian Schilling, Christina Schumacher, Jana Huckauf, Ralf Zimmerman, Renate Horn, Ralf Uptmoor

**Affiliations:** ^1^ Department of Plant Genetics, Institute of Biological Sciences, University of Rostock, Rostock, Germany; ^2^ Department of Agronomy, Faculty of Agricultural and Environmental Sciences, University of Rostock, Rostock, Germany; ^3^ Biomedical Informatics and Engineering Research Laboratory, Syed Babar Ali School of Science and Engineering, Lahore University of Management Sciences (LUMS), Lahore, Pakistan; ^4^ Department of Life, Light, and Matter, University of Rostock, Rostock, Germany; ^5^ Department of Technical and Analytical Chemistry, Institute of Chemistry, University of Rostock, Rostock, Germany; ^6^ Department of Agrobiotechnology, Agriculture, Civil Engineering and Environment, University of Rostock, Rostock, Germany

**Keywords:** *Solanum tuberosum*, drought stress, ethylene biogenesis, phosphorylation, ACC synthase, PP2C phosphatase, 14-3-3 protein

## Abstract

Ethylene plays a critical role in plant development and stress adaptation, with its biosynthesis tightly regulated by the stability of 1-aminocyclopropane-1-carboxylic acid synthase (ACS) proteins. Here, we investigate the potato isozyme StACS3 and its role in modulating ethylene biosynthesis and drought tolerance. StACS3 transcript and protein levels are specifically upregulated under drought stress. In contrast, silencing *StACS3* significantly reduces stress-induced ethylene accumulation and enhances drought resilience, including decreased cell death and increased antioxidant activity. Heterologous expression of *StACS3* in *Arabidopsis thaliana* induces severe developmental phenotypes, such as compact growth, reduced root development, sterility, and accelerated leaf senescence, demonstrating its influence on ethylene-associated processes. Mechanistically, StACS3 is regulated post-translationally through interactions with StPP2C2, a type 2C protein phosphatase that promotes proteasome-mediated degradation, and St14-3-3, a phospho-binding protein that stabilizes StACS3. Mutation and co-expression analysis support the formation of StACS3-StPP2C2 complexes, and silencing StPP2C2 increases StACS3 accumulation and alters its subcellular localization, demonstrating an antagonistic interplay between degradation and stabilization pathways. Collectively, these findings reveal a dynamic post-translational regulatory module that fine-tunes ethylene biosynthesis during drought stress. This study establishes StACS3 as a central node in ethylene-mediated drought response pathways in potatoes, providing mechanistic insights into the balance of protein degradation and stabilization that underlies stress adaptation.

## Introduction

The capacity for ethylene biosynthesis is nearly ubiquitous among plants. However, it is stringently regulated by tissue- and developmental-stage-specific circuitry. Ethylene biosynthesis is modulated by intrinsic developmental signals and various external factors, including pathogens, light, temperature, drought, and flooding (Dubois et al., 2018; Chen et al., 2021; Hartman et al., 2021; Khan et al., 2024). However, stress-induced ethylene activates crosstalk with the regulatory pathways of other hormones. Nevertheless, the interplay between ethylene and abscisic acid (ABA) in regulating stomatal conductance and plant survival is essential under drought conditions (Tanaka et al., 2005; Hasan et al., 2024; Khan et al., 2024).

Ethylene is primarily synthesized through the sequential action of 1-aminocyclopropane-1-carboxylic acid synthase (ACC-synthase, hereafter known as ACS) and ACC oxidase (ACO) enzymes. ACS catalyzes the transformation of S-Adenosylmethionine (SAM) into 1-aminocyclopropane-1-carboxylic acid (ACC). ACC is then converted to ethylene by the catalytic activity of ACO enzymes (Pattyn et al., 2021). Under water deficit conditions, ACS activity is accompanied by an increase in ACC, the direct precursor of ethylene, suggesting that drought induces the *de novo* synthesis of ACS proteins. This in turn enhances ethylene production, with ACS acting as a rate-limiting enzyme (Habben et al., 2014; Zhang et al., 2023). It follows that ACS proteins are critical control points in ethylene biosynthesis.

ACS genes belong to a multigene family in a wide range of plant species. For instance, *Lycopersicon esculentum* (tomato) contains fourteen members, *Oryza sativa* (rice) has six members, and *Arabidopsis thaliana* (*A. thaliana*) comprises 12 members (Gamalero et al., 2023; Khan et al., 2024., Liu et al., 2015). Selected members of the ACS gene family exhibit distinct spatial and temporal expression patterns in response to various developmental, environmental, and hormonal factors (Wang et al., 2005; Gamalero et al., 2023). Accumulating evidence suggests that post-transcriptional regulation is an important aspect of ACS protein turnover and ethylene biosynthesis.

At the protein level, ACSs share a highly conserved N-terminal catalytic core, whereas their C-terminal regions differ in phosphorylation motifs, categorizing them into three distinct classes. Type I ACSs contain sites for phosphorylation by both mitogen-activated protein kinases (MAPKs) and calcium-dependent protein kinases (CDPKs), type II retain only the CDPK site, and type III lacks both (Yoshida et al., 2005; Ludwikow et al., 2014). In *A. thaliana*, ACS1/2/6 are type I, ACS4/5/8/9 are type II, ACS7/11 are type III, and ACS3 is identified as a pseudogene. Type II ACS proteins are regulated via their C-terminal CDPK site, which overlaps with the Target-of-ETO1 (TOE) motif, through interaction with E3 ubiquitin ligase adaptors ETO1, EOL1, and EOL2 (Wang et al., 2004). This recruits the Type II ACS proteins for ubiquitin-mediated proteasome degradation (Wang et al., 2004; Yoshida et al., 2005; Christians et al., 2009)., Hence, the C-terminal phosphorylation reduces their susceptibility to degradation (Joo et al., 2008; Kamiyoshihara et al., 2010; Lee et al., 2021).

In *A. thaliana*, type II ACS5/9 and LeACS3 are recruited by ETO1 for UPS mediated degradation (Yoshida et al., 2005; Christians et al., 2009; Lee et al., 2021), a process at least partially modulated by C-terminal sequences and phosphatases that counteract phosphorylation-based stabilization (Hernandez Sebastia et al., 2004; Hernandez Sebastia et al., 2004; Kamiyoshihara et al., 2010). However, their specific identities and functions have not been fully understood until recently.

The phosphor-binding protein 14-3-3 binds to and stabilizes all three ACS isozyme types in *A. thaliana*, suggesting that phosphorylation is a crucial regulatory step for ACS stability and ethylene biosynthesis. In *A. thaliana* 14-3-3 activity influences ethylene production by destabilizing ETO/EOL proteins while stabilizing ACS proteins in an ETO/EOL-independent manner (Yoon and Kieber, 2013; Lee et al., 2021; He et al., 2022). In potato the roles of 14-3-3 proteins in ethylene regulation remain largely unexplored; however, 11 St14-3-3 isoforms have been identified, with one member shown to interact with StSP6A and a bZIP transcription factor to regulate tuber formation (Teo et al., 2017).

Protein phosphatases such as PP2C and PP2A also contribute to ACS regulation. In *A. thaliana* PP2C phosphatase ABI1 negatively regulates ABA signaling by dephosphorylating ACS6, thereby reducing ethylene production during oxidative stress (Ludwikow et al., 2014). Similarly, PP2A fine-tunes ethylene levels by negatively regulating type I ACS isoforms (AtACS2 and AtACS6) while paradoxically affecting the abundance of type II AtACS5 (Skottke et al., 2011). These findings demonstrate complex isoform-specific regulation of ACS proteins in *A. thaliana*, whereas the functional significance of phosphatase-mediated ACS regulation in potato remains to be investigated.

Given the critical role of potatoes in global food security, enhancing their water-use efficiency through breeding is imperative because the ethylene regulatory network is likely to play a pivotal role. Potatoes are highly vulnerable to drought because of their shallow root systems (Monneveux et al., 2013). Studies have shown that antisense transgenes of StACS4 and StACS5 confer ozone stress tolerance and are transcriptionally activated by stress (Schlagnhaufer et al., 1995, 1997; Sinn et al., 2004).

Furthermore, an association study across 34 potato cultivars, identified allelic variations, implicating StACS3 Soltu.DM.02G027270.1) and StPP2C2 (Soltu.DM.01G021880.1) as candidates for drought tolerance (Schumacher et al., 2021), making them promising targets for breeding drought-resistant varieties. Despite these advancements, the molecular characterization and post-transcriptional regulation of these proteins in potatoes remain poorly understood and require further investigation. Prior to this study, direct evidence of StPP2C interaction with StACS3 was lacking, although comparative insights from *A. thaliana* suggested a potentially similar regulatory mechanism in potatoes.

Additionally, the general role of 14-3-3 proteins in stabilizing phosphorylated targets, including ACS isoforms, has been well established in other plant species (Camoni et al., 2018). However, specific regulatory insights into the interaction between potato St14-3-3 proteins and StACS3, particularly under stress conditions, remain limited. Our study addresses this gap by elucidating the regulatory interactions among StACS3, StPP2C2, and St14-3-3 proteins, revealing a negative feedback mechanism that might contribute to drought tolerance in potato.

In our study, StACS3, a type II ACS isoform, is transcriptionally and post-translationally activated in response to drought stress. StACS3 silencing reduced ethylene production and prolonged plant viability under drought conditions. Conversely, heterologous overexpression in *A. thaliana* results in phenotypic anomalies, including reduced root growth, sterility, and enhanced foliar senescence. Notably, overexpression of *StACS3* leads to elevated hydrogen peroxide (H_2_O_2_) production during drought stress compared to that in wild-type plants.

Functionally, StACS3 directly interacted with StPP2C2, as demonstrated by BiFC and Co-IP assays. Co-expression experiments revealed that StPP2C2 promotes StACS3 degradation, whereas StPP2C2-silencing enhances StACS3 protein stability and accumulation. In contrast, St14-3-3 exhibited a stronger interaction with StACS3, thereby enhancing its stability and recapitulating its phosphorylated state.

Together, these findings support a negative feedback model in which StACS3 stability is modulated by StPP2C2- and St14-3-3-mediated interactions under drought stress. This study advances our understanding of ethylene signaling crosstalk and highlights the potential of this regulatory module to enhance drought tolerance in crop species.

## Materials and methods

### DNA manipulations and plasmid construction

For gene silencing, target-specific fragments of the potato genes *StACS3* (Soltu.DM.02G027270.1), *StPP2C2* (Soltu.DM.01G021880.1), and *StPDS* (Soltu.DM.03G037550.1) were cloned into the pTRV2 vector. For *StACS3*, two sequences (339 and 347 bp) were used for silencing. For phytoene desaturase (PDS), a 372 bp sequence was used. All fragments were first cloned into the pENTR™/SD/D-TOPO^®^ vector (Invitrogen, Thermo Fisher) and subsequently transferred to the pTRV2 silencing vector using LR Clonase™ II (Invitrogen, Thermo Fisher) according to the manufacturer’s protocol. Primer sequences are provided in **Supplementary Table 3**.

For localization studies, the full-length StACS3 coding sequence (CDS) was cloned into the pENTR/D-TOPO vector according to the manufacturer’s instructions. The pEntr-StACS3 clone was sequenced and subsequently subcloned into a pEG104 vector carrying yellow fluorescent protein (YFP) at the N-terminus of the recombination site. LR Clonase II (Invitrogen, Thermo Fisher) was used for subcloning. After sequence verification, Agrobacterium strain GV3101 was transformed with the constructed clones for stable transformation of *A. thaliana* or transient assays in *Nicotiana benthamiana*.

For BiFC, pEntr-ACS3 was subcloned into pEG201-YC and pEG202-YN using LR Clonase II recombination enzyme. The coding sequences for potato StPP2C2 and St14-3-3 (Soltu.DM.11G003450.1) (without the termination sequences) were cloned into pENTRSD/D/Topo. They were then subcloned into a pEG202-YC vector containing the C-terminus of YFP (residues 155–238, designated CYFP) using the Gateway cloning reaction (Schwarzenbacher et al., 2020). ACS3 phosphorylation mutants were engineered to investigate specific serine residues critical for interaction with StPP2C2 by employing the pEG201 vector system. The C1 mutant was generated by substituting the StACS3 serine residues at positions 461 (S461) and 448 (S448) with glycine (G). The C11 mutant involved the substitution of S461 with G, whereas the H1 mutant involved the substitution of S461 with alanine (A) of StACS3. These mutations were introduced via site-directed mutagenesis using the NEBuilder HiFi DNA Assembly Cloning Kit (www.neb.com) following standard protocols. Primers (**Supplementary Table 3**) designed to induce the desired mutations, were utilized in PCR reactions, using the wild-type ACS3-pEG201 construct as a template. The mutated constructs were verified by sequencing.

For the promoter study, a fragment of 1853 bp upstream of StACS3 ORF (-1853 bp) was amplified as promoter StACS3 (pStACS3) from the genomic DNA of the cultivar Desiree. The PCR product was directly digested with BglII and XbaI restriction enzymes. The promoter sequence of pStACS3 was then ligated into the vector pCambia1305.1, which was also digested with BglII and XbaI, followed by gel purification. Competent DH5a cells were transformed with the construct and verified by sequencing. Finally, *Agrobacterium tumefaciens* strain GV3101 was transformed with the constructs for stable plant transformation or transient expression assays into *N. benthamiana* or *S. tuberosum*.

### Plant materials

Stable transformation using the floral dip method was employed to create *A. thaliana* StACS3-YFP transgenic plants (Hu et al., 2019). Transformed plants were selected by spraying with the herbicide Basta (120 mg/L). The StACS3 promoter construct (pACS3) was transformed into *A. thaliana* ecotype Col-0, and transgenic plants were selected using hygromycin (25 mg/L) and cefotaxime (400 mg/L) on ½ MS plates. Desiree tubers were used to grow plants for the ViGS assay, as described by Dobnik et al. (2016). Tobacco (*N. benthamiana)* was used for transient assays. All plants were grown in a greenhouse under natural light or in a growth room at 22 °C with 16/8 h light and dark periods, respectively.

### Sequence alignment and phylogenetic analysis

After construction, all clones were sequenced by Eurofins Genomics (Ebersberg, Germany). The sequences were aligned with the target sequences using Vector Nti (Thermo Fisher Scientific) and MultAlin (http://multalin.toulouse.inra.fr/multalin/). For phylogenetic analysis, full-length ACS protein sequences from potato (StACS), tomato (LeACS), and *A. thaliana* (AtACS) were aligned using the MUSCLE algorithm in MEGA11 (version 11.0; https://mega.nz/). A phylogenetic tree was constructed using the Neighbor-Joining (NJ) method, with evolutionary distances calculated using the p-distance model. Node support was assessed via 1,000 bootstrap replicates. The resulting tree was visualized and annotated using the Interactive Tree of Life (iTOL), with the four ACS subgroups highlighted in different colors (Wang et al., 2020; Shahzad et al., 2023; Liu et al., 2015; Gul et al., 2024).

### Virus-induced gene silencing in *S. tuberosum* and *N. benthamiana* followed by drought stress treatment

For the ViGS assay, plants were grown either in a growth room or a greenhouse. Tubers at 10–14 days post-germination, bearing three to four leaves, were selected for injection with Agrobacterium GV3101 harboring ViGS vectors. Constructs of the respective genes were selected using (https://vigs.solgenomics.net/) and further cross-checked using the VectorNti alignment tool. The bleaching phenotype of PDS silencing was used as a positive indicator of a successful experimental setup. Empty TRV1 vector infiltrations served as controls for all gene silencing experiments. For agroinfiltration, overnight bacterial cultures from single clones were re-cultured in 20 mL LB broth with rifampicin, gentamicin, and kanamycin (100:200:50 mg/L) antibiotics. After 24 h shaking at 28 °C, broth suspensions were centrifuged and washed with MMA (10 mM MgCl_2_, 10 mM MES pH 5.6, acetosyringone 200 µM). Cultures were resuspended in MMA and adjusted to an OD_600_ around one or as described in the text. The cultures were incubated at room temperature for at least 3 h before infiltration. For virus-induced gene silencing (VIGS) experiments, equal amounts of each TRV2 vector carrying a silencing construct (stpds, stacs3, or stpp2c2) were mixed with TRV1 in a 1:1 ratio before agroinfiltration. TRV1, an empty vector (EV), served as the relative control in all experiments.

To investigate plant responses to drought, plants were grown under uniform initial conditions, established by weighing each pot at planting and adjusting the water input after the measurements. Subsequently, the water inputs were reduced to impose continuous 14-day water shortage stress. This method allows for a comparative analysis of plant responses to water limitation.

### Histochemical GUS staining

GUS histochemical staining of transgenic *A. thaliana* plants and transient expression of the StACS3::Gus construct followed a previously described method (Wang et al., 2005). GUS-positive plant tissues were examined using a bright field stereomicroscope (MZFLIII, Leica, Wetzlar, Germany), equipped with a CCD camera (DC 200, Leica, Wetzlar, Germany). Sections mounted on glass slides were observed under an inverted microscope (Leica, DMIRBE, Leica, Wetzlar, Germany), in a bright field and under an ELYRA PS.1 (Carl Zeiss) confocal microscope at 10x, 20x, and 40x magnifications. The GUS-stained plants and tissues shown in this study represent the typical results of at least five independent lines and three biological replicates. For *N. benthamiana* plants, drought stress was applied three days before agroinfiltration and continued for 4–5 days afterwards. All plants were treated with PEG 8000 solution on ½ MS plates at a water potential of -1.2 MPa, following the method described by (Verslues et al., 2006). PEG 8000 was utilized for the GUS assays, while PEG 6000 was employed in all other experiments.

### Protein extraction and fluorometric GUS-assay

Plant protein extraction and assay for GUS activity were performed as described previously (Wang et al., 2005; Mou et al., 2016). Protein concentration of the extracts was determined using the Bradford reagent (Bio-Rad Laboratories, Munich, Germany). Fluorescence was measured using a Microplate Spectrofluorometer (SPECTRAmax GEMINI XS; Biocompare). The excitation and emission wavelengths were 365 nm and 455 nm, respectively. Each assay was repeated three times. The standard curve was generated using serial dilutions of 4-methylumbelliferone (4-MU; 50 to 600 nM) (Sigma-Aldrich Chemie GmbH, Taufkirchen, Germany). Since 4-MU degrades rapidly during storage, fresh solutions were prepared for each use.

### DAB assay and DAB quantification

The production of H_2_O_2_ in ViGSed StACS3 plants was determined by 3,3′-diaminobenzidine (DAB) staining (Daudi and O’Brien, 2012; Kuzniak et al., 2014; Sekulska-Nalewajko et al., 2016). Leaves from drought-stressed and control plants were submerged in 1 mg/mL DAB-HCl solution (pH 3.8). DAB solution was prepared using 0.05% v/v Tween 20 and 10 mM Na_2_HPO_4_. The sample tubes were incubated for 4–6 hours, followed by brief vacuum infiltration. The leaves were then washed with ethanol, acetic acid, and glycerol (3:1:1) wash solution. Falcon tubes containing leaf samples were further boiled in a hot water bath to completely release the chlorophyll. The polymerization of DAB at sites of H_2_O_2_ accumulation generates a brown DAB-polymer.

The DAB-stained leaves were analyzed in ImageJ using a threshold method to isolate the stained areas. The relative mean staining intensity was determined by dividing the intensity of the stained region by the total intensity of the entire leaf area. These ratios were expressed as percentages.

### Ethylene measurements

Tissues from two to three leaves (1 g fresh weight) were excised from the control TRV, ViGSed StPDS and StACS3 plants. Following a 15 min period, the leaves were left uncovered to relieve the ethylene produced by wounding. The tissues were sealed in Cole Parmer head space glass vials with a sample multiplier. After 24h at RT in the dark, 1 ml gas samples were extracted with a syringe and injected into the GC. Ethylene was measured using an Agilent gas chromatography/mass spectrometer system (5977 GC-MSD, Agilent, Waldbronn, DE). A 30m GS-GasPro column (Agilent, Waldbronn, DE) was installed to separate nitrogen and ethylene. Helium at 53cm/sec was used as the carrier gas. Xenon gas with a constant natural background concentration of 87ppbV was used as an internal control for quantification. The MS was operated in single ion monitoring (SIM) mode to detect xenon (Xe; m/z = 131, range 130.70 to 131.70) and ethylene (m/z = 26, range 25.70 to 26.70). Ethylene measurements represent data from at least three biological replicates.

### Ion leakage assay

Leaves from ViGSed StACS3, StPDS, and control TRV-treated plants were collected approximately two week post-drought stress. The leaves were washed twice to remove surface contaminants. All the sample tubes were boiled in a water bath for 5 min. The samples were cooled to room temperature for conductivity measurement. Relative electrolyte leakage was measured by subtracting the values obtained before and after autoclaving (Verslues et al., 2006). The conductivity was measured using a conductivity meter (Vernier Software and Technology, Beaverton, OR, USA).

### Protein localization and bimolecular fluorescence complementation assay

For the localization study, fully expanded four-week-old *N. benthamiana* leaves were infiltrated with Agrobacterium strain GV3101 carrying the pEG104 vector containing the StACS3-YFP cassette. StPP2C2-silenced *N.benthamiana* plants were used for StACS3 agroinfiltration and subsequent protein localization studies. In addition, 4-5-weeks-old plants of the potato cultivar Desiree were used for StACS3-YFP agroinfiltration. MG132, ABA, and PEG 6000 were infiltrated for 3–5 h or as described in the text.

The BiFC assay was employed to assess protein-protein interactions between StACS3, StPP2C2, and St14-3-3 in the epidermal cells of *N. benthamiana* leaves. The constructs used in the assay were as follows: StACS3-YN, where StACS3 was cloned into the pEG201 vector with the N-terminus of YFP (YN); StPP2C2-YC, where StPP2C2 was cloned into the pEG202 vector with the C-terminus of YFP; and St14-3-3-YC, where the potato St14-3-3 protein was cloned into the pEG202 vector with YC. Furthermore C1, C11, and H1 point mutation StACS3 constructs were used for BiFC with StPP2C2. All constructs were co-infiltrated into fully expanded 3–4 weeks old *N. benthamiana* leaves. Confocal images of YFP were taken with an ELYRA PS.1 (Carl Zeiss) confocal microscope. The images were processed using Adobe Photoshop and ImageJ-Fiji-win64 (https://imagej.net/software/fiji/downloads).

### Fluorescence imaging and analysis

Fluorescence images were acquired using a Zeiss ELYRA PS.1 system (Carl Zeiss Microscopy GmbH, Germany), and relative fluorescence intensity was quantified using ImageJ software (NIH, USA). Image analysis was performed by applying a consistent intensity threshold across all images to ensure comparability between control and treatment groups. For quantification, the mean fluorescence intensity of defined regions of interest (ROIs) was calculated.

To normalize the data, the fluorescence intensity of experimental samples was expressed as a ratio relative to the control samples. For improved accuracy, fluorescence values were cross-validated using Zeiss ZEN Blue software, and image scales were calibrated to ensure data consistency and reliability.

### RNA isolation and RT-quantitative PCR

Total RNA was extracted from the cultivar Desiree using the RNeasy Plant Mini Kit (Qiagen, 74904, Hilden, Germany) following the manufacturer’s protocol. Samples were collected from plants subjected to 12–14 days of drought stress, as well as from well-watered control plants. Approximately 100 mg of the leaf tissue was used for each extraction. RNA concentration and purity were assessed using a NanoDrop spectrophotometer (Thermo Fisher Scientific, Dreieich, Germany), and RNA integrity was confirmed by 1% agarose gel electrophoresis. First-strand cDNA synthesis was performed using the QuantiTect Reverse Transcription Kit (Qiagen GmbH, Hilden, Germany), which included a genomic DNA elimination step for accurate downstream quantification.

RT-qPCR was conducted using the Bio-Rad CFX96 Real-Time PCR detection system with SYBR Green. The cycling conditions for StACS3 included an initial denaturation at 98 °C for 2 min, followed by 52 cycles of denaturation at 98 °C for 15 sec, and annealing/amplification at 62 °C for 20 sec. The cycling conditions for the reference gene EF1a, StPP2C2 and other targets were identical, except for the number of cycles, which was reduced to 44. Gene-specific primers (**Supplementary Table 3**) were designed using Integrated DNA Technologies (https://www.idtdna.com/pages/tools). Relative gene expression levels were determined using the 2^-ΔΔCq method, with EF1a serving as an internal control for normalization. All RT-qPCR reactions were performed in technical triplicates and based on at least three independent biological replicates.

### Immunoblot and co-immunoprecipitation

Plants expressing the StACS3-YFP protein were extracted according to (Hamera et al., 2012; Marczak et al., 2020). Protein extracts were prepared in an extraction buffer containing a protease inhibitors cocktail (Roche, Mannheim, Germany). The SDS-PAGE loading dye (1X) was added to each sample and vortexed vigorously. All the samples were boiled for 5 min and centrifuged at 4 °C for 5 min. The supernatants were loaded onto SDS-PAGE gels. StACS3-YFP immunoblotting was carried out using monoclonal anti-GFP antibodies (Santa Cruz Biotechnology, Heidelberg, Germany, sc-32002) at a 1:2500 dilution, followed by anti-rabbit ECL peroxidase secondary antibody (Sigma-Aldrich, Taufkirchen, Germany, NA934VS) at a 1:5000 dilution, and visualized by chemiluminescence using ECL (Amersham Pharmacia Biotech, Braunschweig, Germany). pEG201-ACS3 carries an HA tag and was immunoblotted with anti-HA antibodies (Biozym Scientific GmbH., Hessisch Oldendorf, Germany, B901513) followed by an anti-mouse ECL peroxidase secondary antibody (Sigma-Aldrich, Taufkirchen, Germany, NA931VS) at 1:10000 dilutions. The PEG202 vector carries a Flag tag, and StPP2C2 and St14-3-3 proteins in the BiFC vectors were detected using an anti-Flag (M2, Sigma) antibody.

For Co-IP *N. benthamiana* leaves were agroinfiltrated with pEG201-YN-StACS3, pEG202-YC-StPP2C2, and pEG202-YC-St14-3-3. Equal weights of leaf tissue were harvested and ground in immunoprecipitation (IP) buffer (Hamera et al., 2012; Marczak et al., 2020). Input samples were immunoblotted using anti-HA to detect StACS3 and anti-Flag antibodies for StPP2C2 and St14-3-3. For IP protein-A, beads were washed 5x with wash buffer and incubated with a pre-cleared bait protein-antibody complex for 1–2 hours at 4 °C with gentle rotation. The prey protein extracts (StPP2C2, St14-3-3, and control) were then added to the beads complexes and incubated for an additional 1–2 hours at 4 °C with gentle rotation. After washing the bead complexes from all samples three times with wash buffer, the proteins were eluted using 1X SDS-PAGE loading buffer. The eluted proteins were subjected to SDS-PAGE and subsequent immunoblotting. Wild-type leaf samples were processed as controls to verify the specificity and assess the background signal for co-immunoprecipitation.

### *In-vivo* protein degradation assay

The in-planta degradation assay was performed using Agrobacterium cultures expressing StACS3 and StPP2C2 proteins, each with an initial optical density (OD_600_) of 1, mixed at the following ratios: 1:0.1, 1:0.3, 1:1, and 1:1.3. The resulting mixtures were agroinfiltrated into the leaves of *N. benthamiana*. After 2 days, the leaf samples were harvested, flash-frozen in liquid nitrogen, and ground into a fine powder. Proteins were extracted from equal weights of leaf tissue using extraction buffer (50 mM Tris-HCl, 150 mM NaCl, 1 mM EDTA, 1 mM DTT and 1x protease inhibitor cocktail). Total protein (50 µg) was subjected to SDS-PAGE, followed by immunoblotting. StACS3 was detected using anti-HA antibodies (1:5000), and StPP2C2 was detected using anti-Flag antibodies.

### Root measurements

Root measurements were conducted on plants grown in rhizotrons (24 x 22 cm) using WinRhizo root measurement software (https://regent.qc.ca/assets/winrhizo_about.html). The plants were cultivated in sand and covered with muslin cloth to facilitate root visualization. The experiment was executed in two phases: first, root analysis was performed under well-watered conditions, followed by an assessment under water-deficit conditions induced by 10% PEG 6000. Wild-type and StACS3 transgenic plants were subjected to similar treatments within a shared rhizotron system to ensure comparability.

## Results

### Computational approaches reveal that StACS3 belongs to the type II group of the ACS protein family

**Figure 1:**
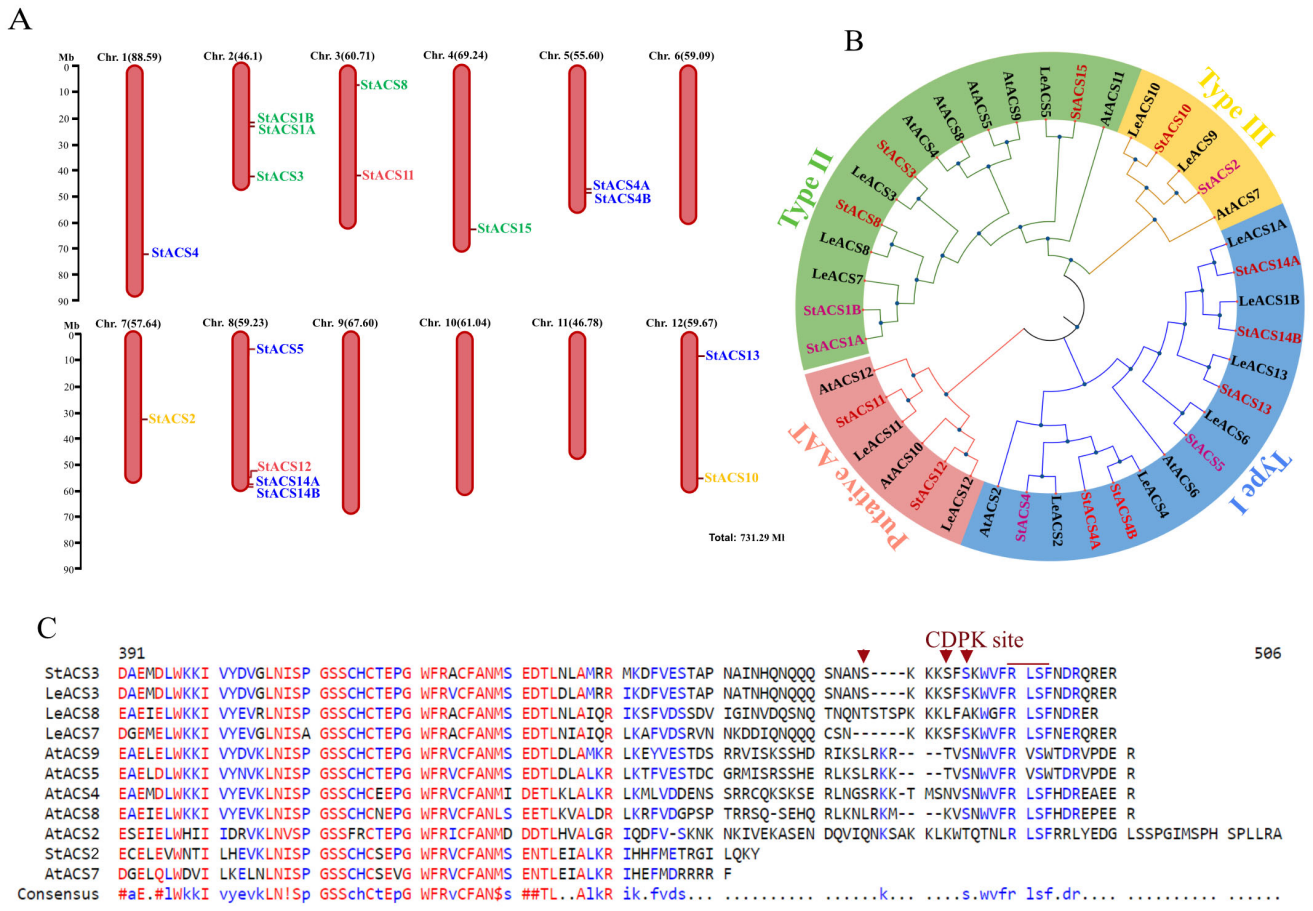
Chromosomal distribution, phylogenetic analysis and multiple sequence alignment of ACS proteins. **(A)** Sixteen StACS genes are mapped across eight of the 12 chromosomes of *S. tuberosum* based on the Phytozome v6.1 genome annotation. Red vertical bars represent chromosomes, with gene positions indicated along the bars. Chromosome lengths (in megabases, Mb) are shown above each chromosome, and the scale bar on the left provides a reference for chromosomal size. StACS3 is located at the distal end of the long arm of chromosome 2. Gene colors indicate classification: Type I (blue), Type II (green), Type III (yellow), and putative aminoacyl transferases (pink). **(B)** Phylogenetic analysis and subgrouping of ACC synthase (ACS) proteins from potato, tomato, and Arabidopsis. A neighbor-joining tree was constructed using the p-distance model based on a multiple sequence alignment of ACS proteins from potato (StACS), *S. lycopersicum* (LeACS), and *A. thaliana* (AtACS). Tree topology was evaluated with 1,000 bootstrap replicates. The ACS family is divided into four distinct phylogenetic subgroups, each highlighted by a different color. Evolutionary analyses were performed using MEGA11 and visualized with iTOL. **(C)** Comparison of the C-terminal domains in ACS proteins. Multiple sequences alignment of StACS3 protein with *A. thaliana* and tomato ACS proteins by MultAlin. Only the C-terminal amino acid sequences of ACS proteins containing putative RLSF phosphorylation sites are presented. Potato Type II ACS (StACS3) and Type III (StACS2) are aligned with tomato Type II (LeACS3, LeACS7, LeACS8), and *A. thaliana* Type II (AtACS4, AtACS5, AtACS8, AtACS9)- Type I (AtACS2)- and Type III (AtACS7). Protein sequences in FASTA format were aligned using default parameters (Corpet, 1988). In the consensus sequence, uppercase letters indicate conserved residues, while lowercase letters represent variable positions. Red denotes high consensus amino acids, while blue and black denote low and neutral consensus sequences, respectively.

1-Aminocyclopropane-1-carboxylic acid synthase (ACS) is encoded by an ethylene-related multigene family and is known to be transcriptionally activated in plants (Park et al., 2021). To explore the diversity and evolutionary conservation of ACS genes in potato, we initially retrieved 17 putatively annotated StACS sequences from the Phytozome v6.03 (https://phytozome-next.jgi.doe.gov/) genome assembly. Sixteen full-length, high-confidence coding sequences were selected based on the presence of complete open reading frames (ORFs), conserved ACS catalytic domains, and absence of premature truncations. Our results showed that these 16 sequences were distributed over eight different chromosomes (**Figure 1A**).

Furthermore, these sequences were validated for functional integrity through multiple sequence alignment and comparative analysis with orthologs from *A. thaliana* and *Solanum lycopersicum* (**Figures 1B, C**). Additionally, five of these twelve sequences had prior experimental support in the literature, particularly in the context of ethylene biosynthesis and transcriptional activation in potato (Destéfano-Beltrán et al., 1995; Schlagnhaufer et al., 1997), strengthening their biological relevance.

Phylogenetic analysis of this curated subset of 16 full-length StACS sequences revealed that the evolutionary counterparts of potato ACS proteins were categorized into three major canonical ACS types (**Figure 1B**). Additionally, phylogenetic analysis revealed that StACS3 is a close homologue of the tomato genes LeACS3, LeACS7, and LeACS8, as well as *A. thaliana* genes AtACS4, AtACS5, AtACS8, and AtACS9 (**Figure 1B**). In addition to the phylogenetic findings, the computed molecular weights of potato ACS proteins ranged between 50 to 60 kDa, which is consistent with ACS proteins from other plant species (Kende, 1993; Liu et al., 2015).

Amino acid sequence alignment of the putative StACS3 with its tomato (LeACS) and *A. thaliana* (AtACS) orthologs revealed conserved motifs, including a CDPK phosphorylation site at serine residues, along with the consensus motif “WVFRLSF” followed by an R/D/E-rich region in the hypervariable C-terminal domain Notably, the absence of an extended amino acid sequence downstream of the “RLSF” motif, present in type I AtACS2, supports the classification of StACS3 as a type II ACS (**Figure 1C**).

### Drought stress significantly upregulates *StACS3* transcript accumulation

**Figure 2:**
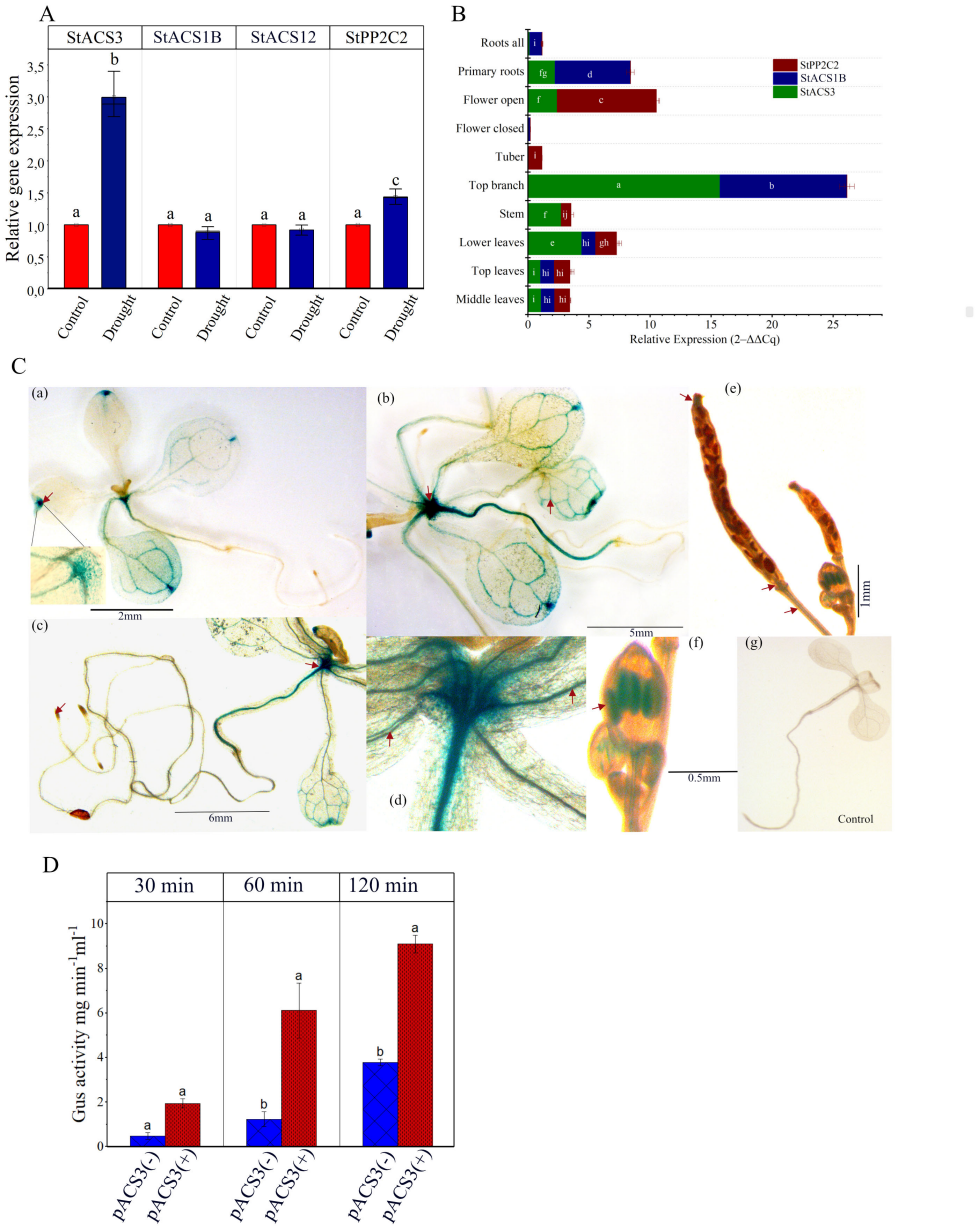
Relative gene expression and pACS3::GUS analysis in developing *A. thaliana* transgenic plants. **(A)** Five- to six-week-old potato plants were subjected to water stress for 12 to 14 days. Transcript levels of *StACS3*, *StPP2C2*, *StACS1B*, and *StACS12* were assessed using RT-qPCR. The *EF1α* gene was used as an internal reference to normalize gene expression. Bar graphs represent relative gene expression levels in control and drought-treated samples. Data are shown as means ± standard error from at least three independent experiment with at least 3 biological replicates. Statistical analysis was performed using two-way ANOVA followed by Tukey’s *post hoc* test. Different letters above bars indicate significant differences (p < 0.05) between treatment and genes. **(B)** Tissue-specific expression profiles of *StACS3, StACS1B*, and *StPP2C2* in potato are shown. Stacked bars representing mean relative transcript levels (2–ΔΔCq) across the following tissues: middle leaves, top leaves (reference tissue), lower leaves, stem, top branch, tuber, closed flowers, open flowers, primary roots, and whole roots. Data were normalized to *EF1α* and are based on three independent experiments, each with a minimum of 3 biological replicates, each measured in triplicate qPCR reactions. Error bars represent standard error (SE). Two-way ANOVA identified significant effects of gene, tissue, and their interactions (p < 0.0001). Tukey’s *post-hoc* test (p < 0.01) was used for pairwise comparisons; different letters indicate significant differences. Letters are not shown for tissues with expression levels below the detection limit. **(C)** 2–3 weeks-old light-grown pACS3::GUS plants were treated with PEG (-1.2 MPa water potential) for 36 hours. GUS expression is shown in leaf hydathodes (a), shoot apical meristem (b), vascular tissues (b, d), anther (stamens) (f) and post-pollinated siliques (style, internodes) (e) (see arrowheads). (a, b) are presenting transgenic lines 6 and 4, respectively. Root tips did not exhibit any expression, as shown in (c). Plants subjected to no stress did not demonstrate GUS expression, as illustrated in (g). The staining patterns shown are representative of observations from at least five independent transgenic lines, with 8–10 plants analyzed per line across three independent experiments. **(D)** Quantitative fluorometric assay of GUS activity in *N. benthamiana* leaves infiltrated with pACS3::GUS. Four-week-old leaves were infiltrated with *Agrobacterium tumefaciens* (OD_600_ = 1.0) carrying the pACS3::GUS construct. Samples were collected four days post-infiltration and subjected to PEG-induced drought stress for 12 hours. Blue bars indicate GUS activity from leaf discs incubated on ½ MS plates with buffer only (pACS3(-)), while red bars represent discs incubated on PEG-treated ½ MS plates (pACS3(+)). Data are presented as mean ± SD from three independent biological replicates. Each biological replicate comprises a pool of six leaf discs, with measurements taken in three technical replicates.

To investigate the molecular basis of the drought-specific response of StACS3, we analyzed its transcript levels in *S. tuberosum* cv. Desiree and two other drought-resistant and drought-sensitive potato varieties (Sprenger et al., 2015; Schumacher et al., 2021). Our results indicated that *StACS3* has minimal expression under optimal growth conditions. However, under drought stress, *StACS3* becomes transcriptionally activated, showing a 2.99-fold upregulation (p < 0.0001, Tukey’s HSD test) compared to the well-watered control. The expression of *StACS1B* and *StACS12* remained unchanged. In contrast, *StPP2C2* expression was also significantly elevated, showing a more modest 1.45-fold upregulation (p = 0.031) under drought conditions (**Figure 2A**). Similar to Desiree, a significant upregulation of *StACS3* was observed in both drought-sensitive and drought-resistant potato varieties, with fold-increases of 2.28 and 2.37, respectively (**Supplementary Figure 1A**). These results suggest that transcriptional regulation is the primary mechanism, although the potential influence of altered mRNA stability cannot be ruled out completely. To evaluate tissue-specific expression of *StACS3*, we compared its expression in different plant tissues. RT-qPCR results revealed the highest *StACS3* expression in top branches, followed by the etiolated lower leaves, stems, and proximal parts of primary roots (**Figure 2B**).

To further explore the stress-responsive regulation of StACS3, a *de novo* search using PLACE (http://www.dna.affrc.go.jp/PLACE/) identified multiple cis-regulatory stress-responsive elements in its promoter, including ABRE, MYC, MYB, ERF, and DOF binding sites (**Supplementary Table 1**). Notably, the DOF motif (TAAAGSTKST), previously associated with guard cell-specific gene expression (Plesch et al., 2001; Cominelli et al., 2011), suggests a potential role of StACS3 in stomatal regulation under stress.

To functionally assess stress responsiveness, we generated *A. thaliana* transgenic lines harboring the StACS3::GUS construct and analyzed tissue-specific GUS expression under drought stress conditions. Although *A. thaliana*-specific transcription factors may influence promoter activity, this heterologous system offers a tractable model for evaluating conserved stress-responsive regulatory elements. Upon drought analog-PEG-induced drought stress, StACS3 promoter activity was upregulated in the cotyledons, primary leaves, shoot apex, hypocotyl, root vascular tissues, and leaf hydathodes. In contrast, no GUS activity was detected under optimal growth conditions (**Figure 2C**; **Supplementary Figure 1**; **Supplementary Table 2**). Additionally, cold and heat stress treatments led to a substantial increase in GUS expression driven by the StACS3 promoter (**Supplementary Figure 1D**).

It is important to note that StACS3 expression was not immediate; instead, it required prolonged stress exposure, typically overnight, or severe PEG-mediated stress conditions (-1.2 MPa). Following these observations, transient expression assays were also conducted via agroinfiltration of *N. benthamiana* leaves, followed by PEG treatment to simulate drought stress. The fluorometric GUS assay revealed a significant increase in StACS3 expression under stress conditions (**Figure 2D**). Consistent with these results, histochemical GUS staining of the infiltrated leaves confirmed StACS3 expression in specific tissues, including vascular bundles, hydathodes and trichome (**Supplementary Figure 1C**). These results collectively support the hypothesis that StACS3 is transcriptionally activated in response to drought stress and that its expression is spatially localized within key tissues involved in water regulation and stress responses.

### Silencing of StACS3 enhances drought tolerance and reduces stress-induced ethylene and ROS (H_2_O_2_) production in potato

**Figure 3:**
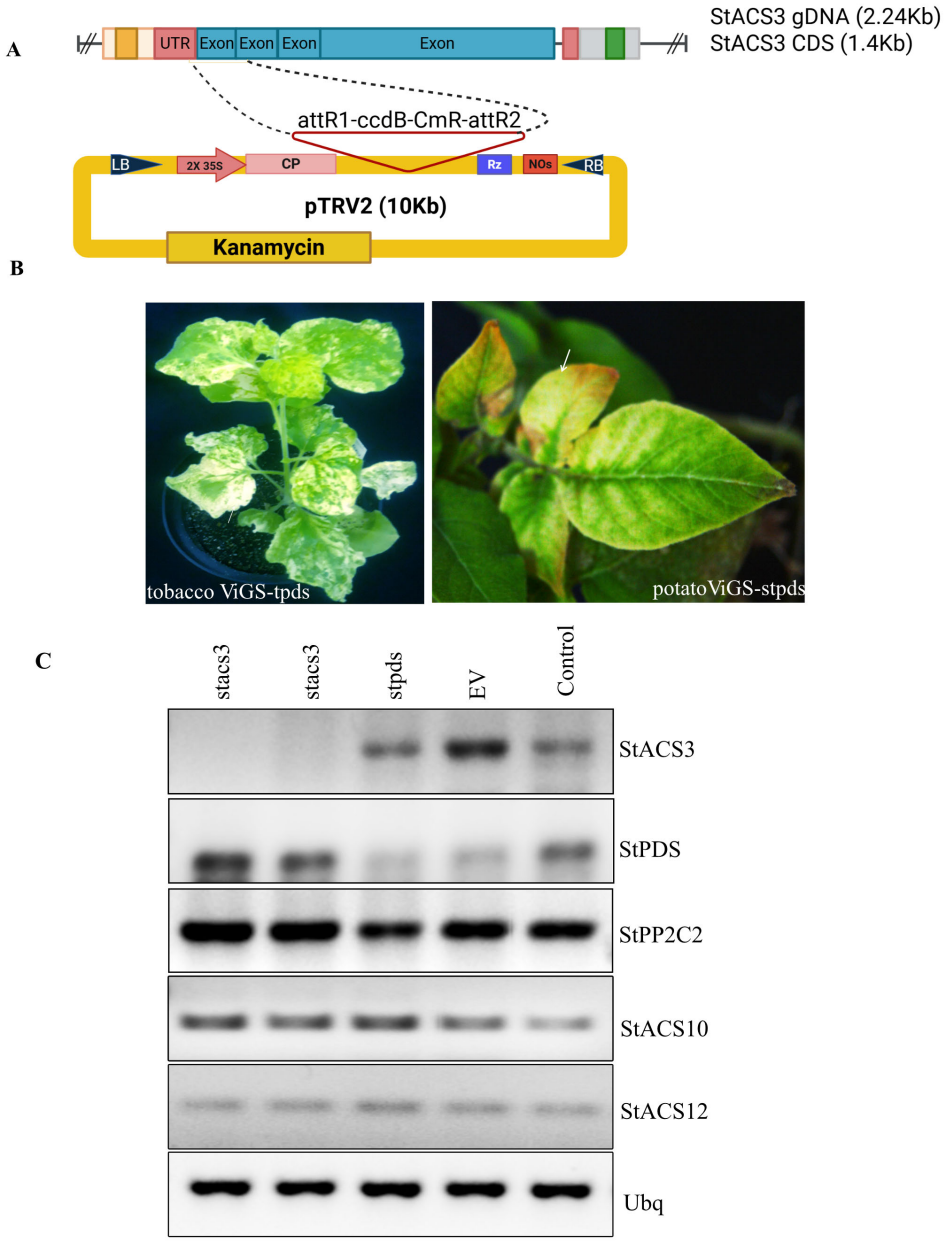
Overview of the cloning strategy, assessment of silencing efficiency, and analysis of gene expression in virus-induced gene silencing (ViGS) experiments. **(A)** Schematic representation of *StACS3* fragment cloning into the TRV1 vector used for virus-induced gene silencing (ViGS). **(B)** Gene silencing efficiency was visualized using the *StPDS* marker gene, which produces a characteristic photobleaching phenotype. Representative images are shown for *N. benthamiana* (left) and *Solanum tuberosum* cv. Desiree (right). **(C)** Semi-quantitative RT-PCR confirms silencing of *StACS3* and *StPDS* in potato. Transcript levels were normalized to *ubiquitin* (*Ubq*) as an internal control. Only verified silenced plants were used for downstream analyses.

To elucidate the function of StACS3 in response to drought stress, we employed Virus-Induced Gene Silencing (ViGS) to transiently knock down StACS3 expression in potato (**Figure 3A**). VIGS was selected as the preferred reverse genetics tool due to its rapid implementation and cultivar-specific efficacy, particularly in *Solanaceae* species where it has been extensively validated (Dobnik et al., 2016; Courdavault et al., 2020). Initial optimization of the VIGS system was conducted across multiple Solanum species, including *three S. tuberosum* cultivars and *Solanum venturii* (Dobnik et al., 2016), using PDS as a visual marker for silencing efficiency. Among the tested plants, the potato cultivar Desiree exhibited the most consistent and robust silencing phenotype (**Figure 3B**) and was thus selected for all further experiments. Silencing of StACS3 in Desiree led to a significant decrease in transcript levels, as verified by semi-quantitative RT-PCR analysis (**Figure 3C**), validating the effectiveness of the VIGS approach in this study (**Figure 3A**).

**Figure 4:**
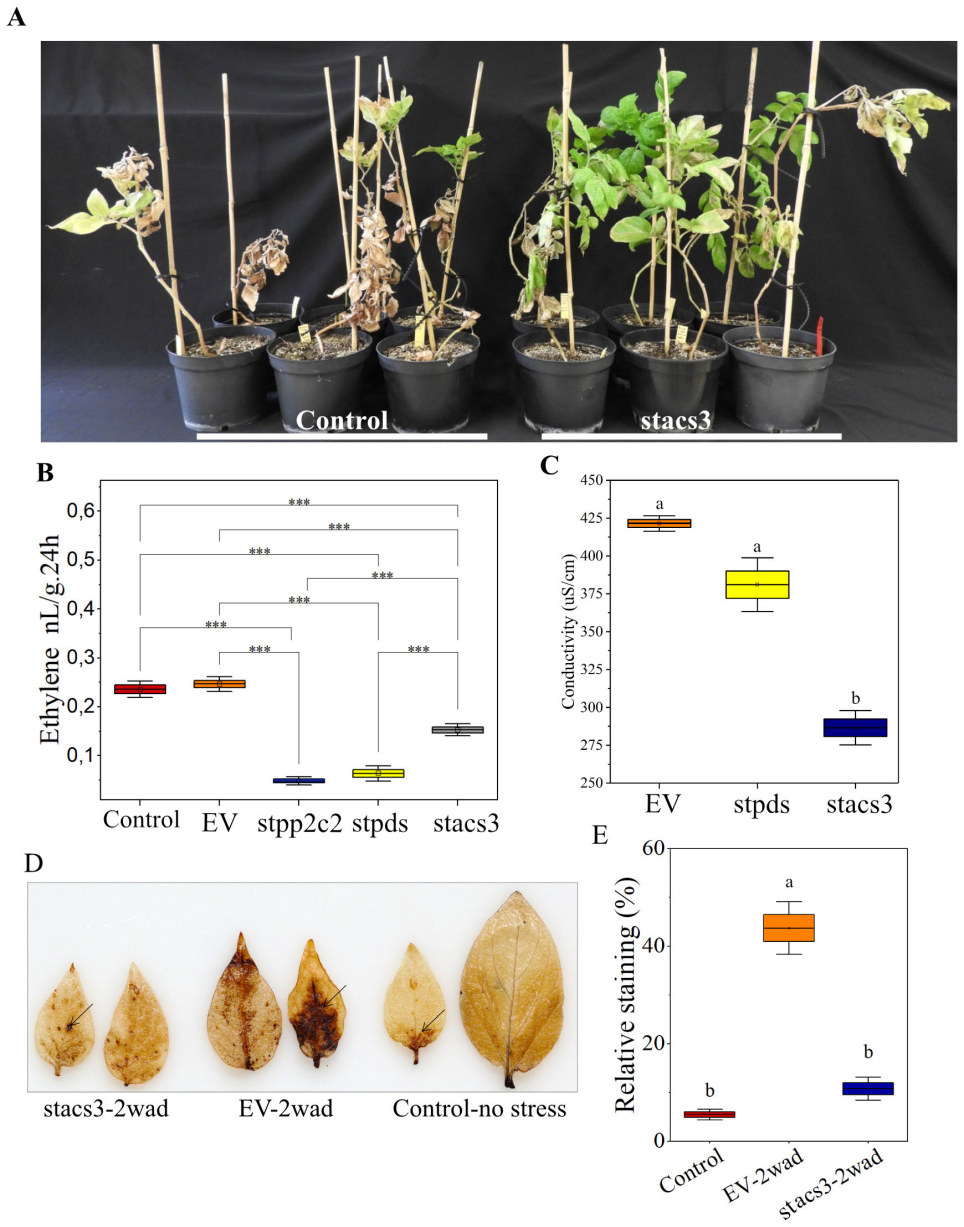
*StACS3* transient gene silencing in potato enhances drought tolerance, reduces ethylene biogenesis, and mitigates reactive oxygen species (ROS) accumulation. **(A)** Potato plants were transiently silenced for the *StACS3* gene using the TRV vector at 10 days of post-germination. At 7–8 weeks post-virus-induced gene silencing (ViGS), the plants were subjected to drought stress for 14 days. Plants silenced for *StACS3* (*stacs3*) exhibited a drought stress tolerance phenotype compared to control plants inoculated with the TRV empty vector (Control). For consistency, the second and third top branches were assessed for drought stress phenotype. A total of eight to ten plants were analyzed per biological replicate, and the drought stress experiment was independently repeated at least four times (n = 4). **(B)** Ethylene levels were measured in *stacs3*-, *stpds*-, and *stpp2c2*-silenced plants, as well as empty vector (EV) and wild-type control plants after 2 weeks of drought stress. Leaf samples from different branches were pooled and sealed in vials, and ethylene production was quantified after 24 hours (n = 4). A one-sample t-test was performed, showing significant differences (p < 0.001) across all tested silenced groups compared to controls (*stacs3 vs* Control and EV, *stpds* and *stpp2c2 vs* Control, and EV), indicated by ***. **(C)** Electrolyte leakage was measured from leaf disks taken from *stacs3*, *stpds*, and empty vector (Control) plants after 2 weeks of drought stress. Electrolytic conductivity significantly increased in control plants following drought stress, whereas the increase was limited in stacs3 plants compared to both control and *stpds* plants. Data are presented as mean ± SEM of three independent biological replicates (n = 3). For each biological replicate, the mean value of three technical measurements is shown. Means were compared by one-way ANOVA followed by Tukey’s *post-hoc* test. Boxes not sharing a common letter are significantly different (p < 0.05). Exact p-values for all pairwise comparisons are provided in **Supplementary Table 5**. **(D)** Hydrogen peroxide production was visualized in *StACS3*-silenced (*stacs3*) and empty vector (EV) control plants after two weeks of drought treatment (2 wad). Leaves from wild-type plants grown under well-watered conditions (Control, no stress) served as the negative control. The images shown are representative of results from three independent experiments. **(E)** Hydrogen peroxide accumulation was quantified using ImageJ, with bars representing the relative stained area (%). Data are presented as mean ± SEM from three independent experiments, each including three to five biologically independent plants per genotype. Statistical significance was assessed by one-way ANOVA followed by Tukey’s *post-hoc* test (p < 0.05); different letters indicate significant differences. Exact p-values are reported in **Supplementary Table 5**.

StPDS-silencing in parallel demonstrated reduced transcripts with the expected bleaching phenotype. To rule out cross-silencing within the ACS gene family, we annotated the ViGS target region and performed semi-quantitative PCR. This analysis confirmed that silencing was specific to StACS3, with other ACS homologs maintaining the wild-type expression levels (**Figure 3C**). Eight weeks post-ViGS treatment, the StACS3-silenced plants were subjected to drought stress. These plants exhibited enhanced drought tolerance compared to those administered the TRV empty vector (**Figure 4A**), establishing a genetic link between StACS3 silencing and improved drought tolerance in potatoes.

Given the elevation of StACS3 transcripts during drought stress (**Figure 2**) and the observed drought tolerance in StACS3-silenced plants (**Figure 4A**), we explored the correlation between StACS3 silencing and stress-induced ethylene production. Quantitative assessments of ethylene revealed that StACS3 silencing resulted in a marked reduction in stress-induced ethylene levels under drought conditions when compared with the wild-type and TRV1 empty vector (EV) controls. Similarly, the other silencing controls, StPDS- and StPP2C2-, also influenced the production of stress-induced ethylene in potato plants (**Figure 4B**).

Furthermore, transient gene silencing indicated a significant decrease in ion leakage and reactive oxygen species (ROS), particularly H_2_O_2_, in StACS3-silenced plants under drought stress compared to controls (**Figures 4C–E**). These observations highlight the inhibitory regulatory role of StACS3 in the drought stress response, including the modulation of ethylene synthesis and ROS generation.

### StACS3 overexpression in heterologous *A. thaliana* results in plant growth anomalies and enhanced antioxidant production

**Figure 5:**
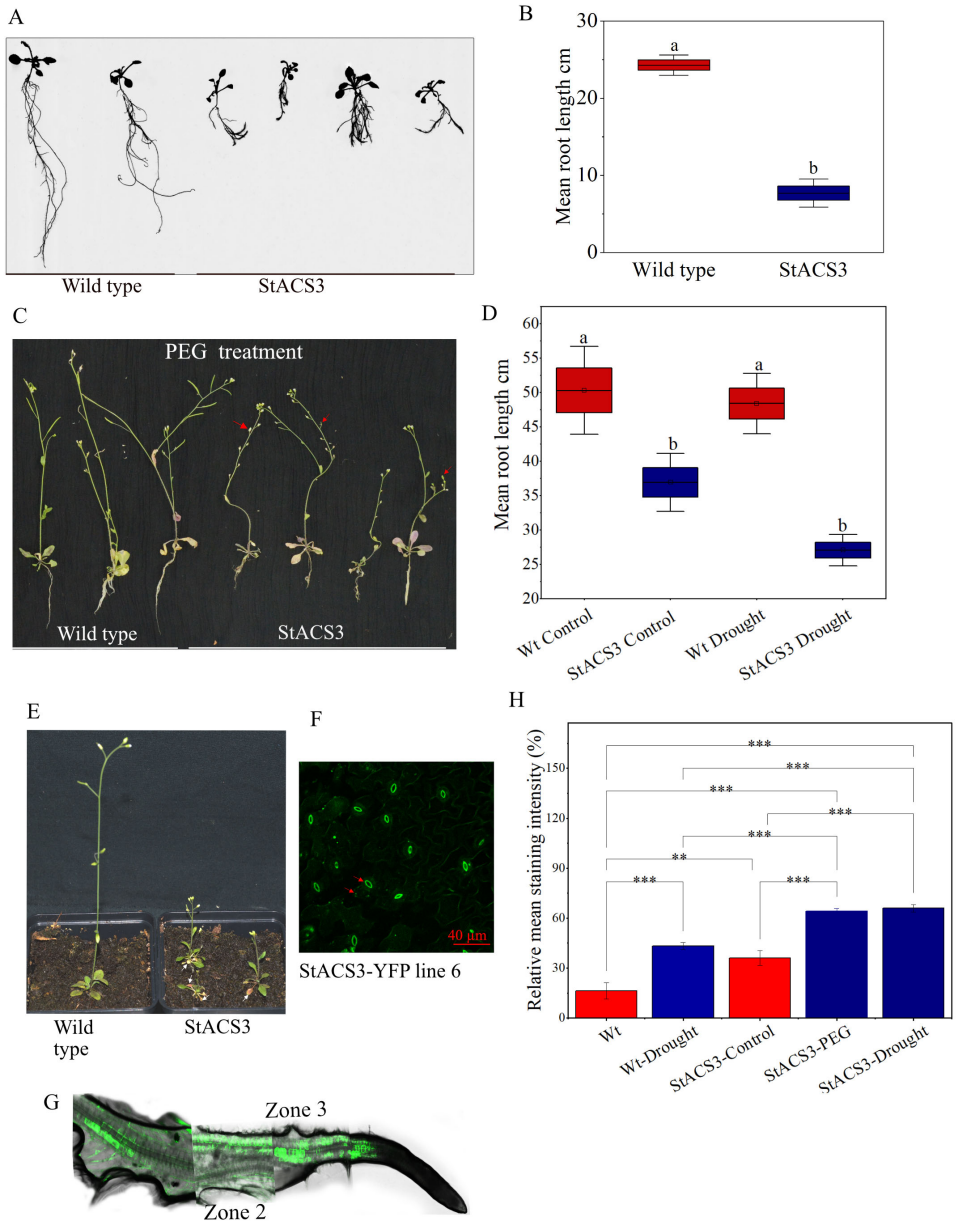
*StACS3* overexpression in *A. thaliana* shows phenotypic anomalies, reduced root growth and increased H_2_O_2_ (ROS) production. **(A)** Root phenotype of *A. thaliana* overexpressing *StACS3* after 7 days of transfer to rhizotrons. Transgenic *StACS3* plants exhibited reduced root lengths compared to wild-type controls. **(B)** Root length analysis displayed significant differences between wild-type controls and *StACS3*-overexpressing transgenic plants. Total root length was measured with WinRhizo software. Statistical analysis was performed using one-way ANOVA followed by Tukey’s test to determine significance. The graph was created using OriginPro, illustrating the measured root lengths. **(C)** Phenotypic comparison of wild-type and *StACS3* transgenic *A. thaliana* plants under drought stress induced by PEG 6000 treatment. Transgenic plants displayed reduced height, root lengths, and sterile inflorescences (indicated by arrowheads) compared to wild-type plants. **(D)** Root lengths of drought-stressed plants measured with ImageJ, highlighting the differences in response to PEG treatment between wild-type and *StACS3* transgenic plants. **(E)** Observation of senescence in the lower leaves of *StACS3*-overexpressing transgenic plants (white arrowheads indicating senescing leaves). **(F)** Localization of StACS3-YFP protein expression, predominantly in stomata and in the form of doted punctuates. **(G)** Localization of StACS3-YFP in transgenic *A. thaliana* roots. StACS3-YFP localizes to the cell membrane and is distributed throughout the vascular tissues, particularly in the dividing and differentiated zones (zones 2 and 3, respectively) of the roots. The apparent patchiness in the figure is due to the technical constraints of confocal microscopy, which cannot capture the entire root in a single scan. To visualize the complete root structure, multiple optical sections were acquired and subsequently assembled into a composite image. **(H)** Relative mean staining intensity of StACS3-YFP transgenic plants under drought (6–8 days) and PEG 6000 (10%) treatment. StACS3 transgenic plants exhibited increased DAB staining intensity under stress, as assessed using the relative intensity threshold method, which calculates the total leaf intensity divided by the absolute intensity and then multiplies it by 100 to express the value as a percentage. Staining intensities were quantified with ImageJ software. Blue bars represent stressed plants, and red bars represent the corresponding controls. Statistical analysis was performed using one-way ANOVA followed by Tukey’s test to determine significance.

To elucidate the role of StACS3 in plant physiology and stress tolerance *in vivo*, we transformed StACS3-YFP into heterologous *A. thaliana*. The transgenic plants exhibited pronounced phenotypic anomalies, including dwarfism, premature leaf senescence, and significantly reduced root growth (**Figures 5A–E**). Under drought stress, root length remained notably shorter than that of control plants (**Figures 5C, D**). Additionally, the transgenic plants displayed sterility, further emphasizing the deleterious effects of StACS3 overexpression on overall plant development and reproductive viability (**Figure 5C**). Given that potato’s agronomically crucial organs are stolons and tubers, which are absent in *A. thaliana*, the physiological relevance of this sterility phenotype to potato may be limited. Conversely, the leaf and root phenotypes are suggesting that StACS3 overexpression disrupts conserved cellular processes, likely through ethylene-mediated signaling. These findings highlight the crucial role of tightly regulated StACS3 activity in maintaining normal growth and stress sensitivity in plants. Interestingly, in transgenic plants, the StACS3-YFP fluorescence signal was predominantly observed in the thick membrane of guard cells, along with punctate in the cell membrane and within the cells of leaves and flower petals (**Figure 5F**, **Supplementary Figure 2**). Furthermore, in the roots, the fluorescence signal was prominent in the dividing and differentiation zones (zones 3 and 2, respectively), whereas no signal was detected in the meristematic zone (**Figure 5G**).

Analysis of DAB-stained leaves using ImageJ revealed a significant increase in staining intensity of PEG-treated and drought-stressed StACS3 transgenic plants compared to controls in both well-watered and water-stressed conditions (**Figure 5H**). This elevated DAB staining intensity suggests a higher level of hydrogen peroxide (H_2_O_2_) accumulation in StACS3-expressing plants under stress, indicating that StACS3 overexpression may experience increased oxidative stress in response to drought.

### Drought stress and proteasome inhibition modulate StACS3 protein

**Figure 6:**
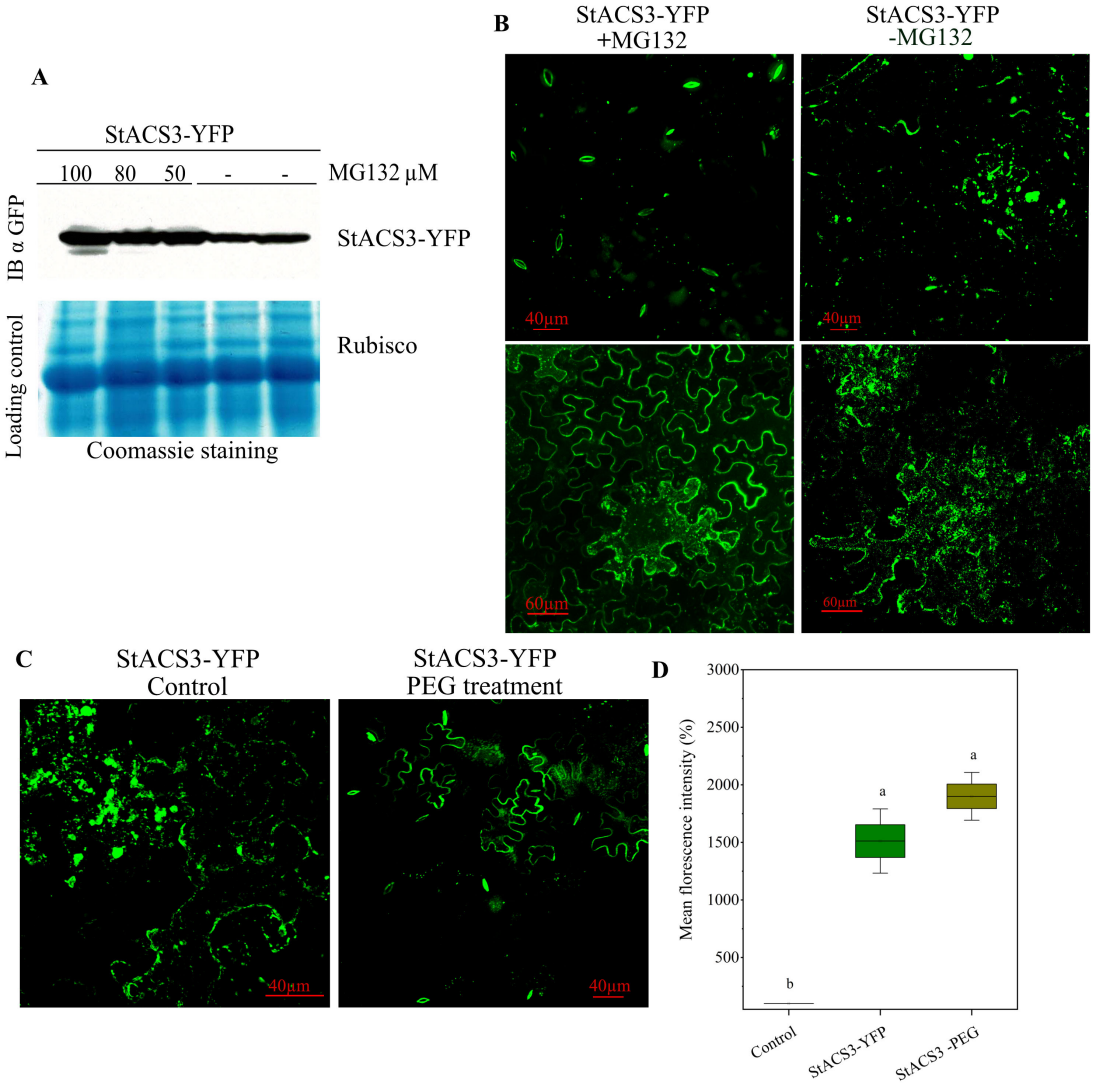
Expression and localization of StACS3 protein following proteasome inhibitor and PEG-induced stress treatment. **(A)** *N. benthamiana* leaves were agroinfiltrated with StACS3-YFP at an optical density of 0.6. Two days post-inoculation, MG132 was introduced into StACS3 pre-infiltrated leaves at concentrations of 100, 80, and 50 µM. Following 4–6 hours of MG132 treatment, samples were collected. Immunoblots were analyzed using anti-GFP antibodies (top panel). Coomassie blue staining was used to detect ribulose-1,5-bisphosphate carboxylase/oxygenase (Rubisco) subunits, serving as a loading control. **(B)** StACS3-YFP agroinfiltrated leaves were subjected to MG132 treatment after 2–3 dpi. MG132 treatment (left panels) showed YFP localization occasionally in guard cells, but primarily at the cell membrane, compared to mock treatment (right panels). **(C)** Leaves expressing StACS3-YFP for 48 hours were treated with drought analog PEG (15%) by agroinfiltration, and samples were analyzed after 4–6 hours. Z-stack images were captured using a Zeiss microscope with uniform brightness. **(D)** Mean fluorescence intensity of StACS3-YFP and StACS3-YFP PEG-treated samples was quantified using ImageJ. Relative intensities were measured with the negative control (empty vector) serving as the background signal. Data were analyzed using one-way ANOVA followed by Tukey’s *post hoc* test (p < 0.05). There was no statistically significant difference between PEG-treated and untreated StACS3-YFP samples, despite a numerical increase in mean fluorescence.

To investigate the regulatory pathway of the StACS3 protein, we first examined its expression and distribution in response to proteasome inhibition and drought-analog PEG treatment. Proteasome inhibition was performed by infiltrating leaves expressing StACS3-YFP at varying concentrations of the proteasome inhibitor MG132. Immunoblot analysis revealed significant accumulation of StACS3 protein in MG132-treated tissues compared to the control, supporting the involvement of the UPS in regulating StACS3 turnover (**Figure 6A**).

StACS3-YFP was primarily localized as punctate and bead-like structures within the membrane and cytoplasm. However, following proteasome inhibitor (MG132) treatment, StACS3-YFP accumulated substantially at the cellular membrane and in the thickened cell walls of stomata, indicating protein stabilization and a shift in its subcellular localization (**Figure 6B**). In *N. benthamiana* StACS3 was predominantly associated with the plasma membrane; however, occasional shifts to stomatal localization were observed. This stomatal localization, however, occurred more frequently in transgenic *A. thaliana* plants and *S. tuberosum* plants (**Figure 6**). Together, these observations indicate that UPS-mediated degradation is a key regulatory mechanism governing the stability and subcellular distribution of StACS3.

Additionally, we investigated the localization of StACS3 under PEG-induced water-deficit conditions. Confocal microscopy revealed that StACS3-YFP was predominantly localized to the plasma membrane, mirroring the distribution observed following MG132 treatment (**Figure 6C**). This suggests a potential synergistic effect of drought stress and UPS-mediated regulation in orchestrating StACS3 stabilization and spatial distribution (**Figure 6C**). Under drought stress, StACS3-YFP exhibited consistent localization along the cell membrane and within guard cells, whereas in control plants, a punctate and scattered distribution was predominant. This differential redistribution suggests that the stress-induced configuration of StACS3 corresponds to its transition to a more functionally active state. Although fluorescence intensity measurements revealed no significant difference in relative mean fluorescence between control and drought-stressed samples (**Figure 6D**), this indicates that the overall fluorescence intensity does not necessarily reflect StACS3’s functional dynamics. Instead, stress-induced stabilization and membrane localization of StACS3 reinforce the importance of post-translational modifications, potentially mediated by phosphorylation, in regulating its activity during drought conditions.

### The StACS3 protein is subject to tight regulation by the StPP2C2 protein phosphatase

**Figure 7:**
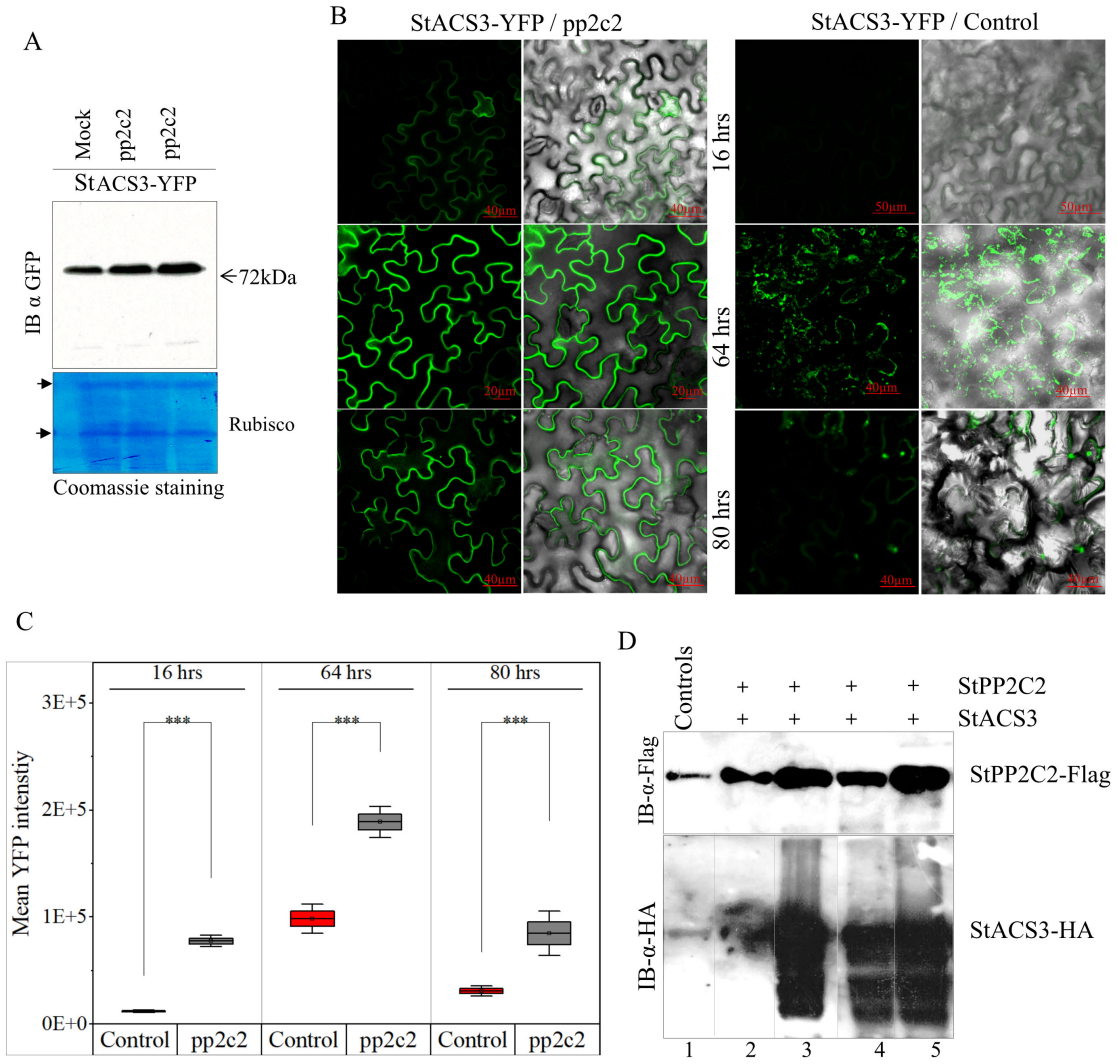
StPP2C2 silencing upregulates StACS3 protein and co-expression degrades StACS3. **(A)** Immunoblot of StACS3-YFP in StPP2C2-silenced plants. Leaves from PP2C2-silenced (pp2c2) plants were infiltrated with StACS3-YFP and harvested 48 hours post-transfection. Immunoblotting with anti-GFP antibodies showed increased StACS3-YFP accumulation in pp2c2 plants compared to control mock-treated plants. The 72kDa band corresponds to StACS3, with Rubisco subunits as loading controls. **(B)** PP2C2-silenced plants were subjected to agroinfiltration with YFP-tagged StACS3 and subsequently visualized at 16, 64, and 80 hours post-infiltration. Fluorescence images captured with confocal microscope (left panels) are overlaid with DIC images (right panels) to provide structural context. Scale bars are provided on the panels. Z-stack images were acquired at 10 µm intervals and visualized as maximum-intensity projections. Due to high signal intensity, the 64-hour StACS3-YFP/pp2c2 image is presented as an average intensity projection. **(C)** The mean YFP fluorescence intensity of StACS3-YFP samples from **(B)** was quantified utilizing ImageJ software. A threshold method was employed to measure YFP fluorescence, with the background signal subtracted accurately. Fluorescence intensity was normalized across all samples, and statistical analysis was conducted using one-sample t-tests. Asterisks (***) denote significant differences at p < 0.001. Error bars represent standard errors derived from three independent biological replicates. **(D)** The degradation of StACS3 was assessed through co-infiltration with varying concentrations of StPP2C2 into *N. benthamiana* leaves. Samples were collected 48 hours post-transfection, and StACS3 levels were analyzed by immunoblotting using an anti-HA antibody, while StPP2C2 was detected using an anti-FLAG antibody. Minimal StACS3 degradation was observed at the lowest concentration of StPP2C2 (1:0.1 ratio, lane 2), with substantial degradation occurring at exceeding concentrations (1:0.3, 1:1, and 1:1.3 ratios in lanes 3-5), as evidenced by proteolyzed StACS3 forms. Control lanes show the individual expression of each protein in leaf tissues.

Our findings demonstrate that StPP2C2-silencing in potato significantly increased the accumulation of StACS3 protein, as shown by western blot analysis (**Figure 7A**). Additionally, in plants with suppressed StPP2C2, the accumulation signal of YFP-tagged StACS3 was markedly enhanced, suggesting potential alterations in protein stability and subcellular localization. Temporal expression analysis further revealed that StACS3 signal intensity and localization were sustained for an extended duration in StPP2C2-silenced plants. The accumulation peaked at 64 hours post-infiltration (hpi) and remained detectable up to 80 hpi, indicating enhanced protein stability (**Figure 7B**). To ensure reproducible comparison of fluorescence signals across samples, a standardized quantification approach was employed using ImageJ threshold analysis, adapted from protocols established in both medical and plant cell biology (Zhang et al., 2020a). This analysis revealed a statistically significant increase in YFP signal intensity in StPP2C2-silenced plants compared to that in the controls, supporting enhanced StACS3 accumulation under reduced phosphatase activity (**Figure 7C**).

To further investigate the post-translational regulation of StACS3 by StPP2C2, a heterologous in planta degradation assay was performed by transiently co-expressing StACS3 and StPP2C2 at varying ratios in *N. benthamiana* leaves. Immunoblot analysis revealed that StACS3 underwent significant post-translational modifications in response to increasing concentrations of StPP2C2. Minimal degradation was observed at the lowest StPP2C2 concentration (1:0.1), whereas substantial degradation occurred at higher concentrations, resulting in pronounced StACS3 proteolysis (**Figure 7D**). Concurrently, lower molecular weight bands were detected as diffuse smears and a laddering pattern, indicative of degradation intermediates or proteolytic processing of StACS3 (lower panel, **Figure 7D**). Collectively, these findings suggest that the enhanced stability and altered localization of StACS3 in StPP2C2-silenced plants result, at least in part, from modulation of post-translational processes. While the use of a pharmacological MG132 and the observed degradation patterns strongly support the involvement of the UPS in StACS3 turnover, future studies utilizing genetic UPS markers (e.g., dominant-negative proteasome mutant PBA1) could further substantiate this degradation pathway. This evidence supports the critical role of StPP2C2 in modulating StACS3 turnover, potentially through mechanisms involving protein dephosphorylation that ultimately target StACS3 for UPS-mediated degradation.

### StACS3 interacts with StPP2C2 and St14-3-3 proteins, and its serine mutants retain binding to StPP2C2

**Figure 8:**
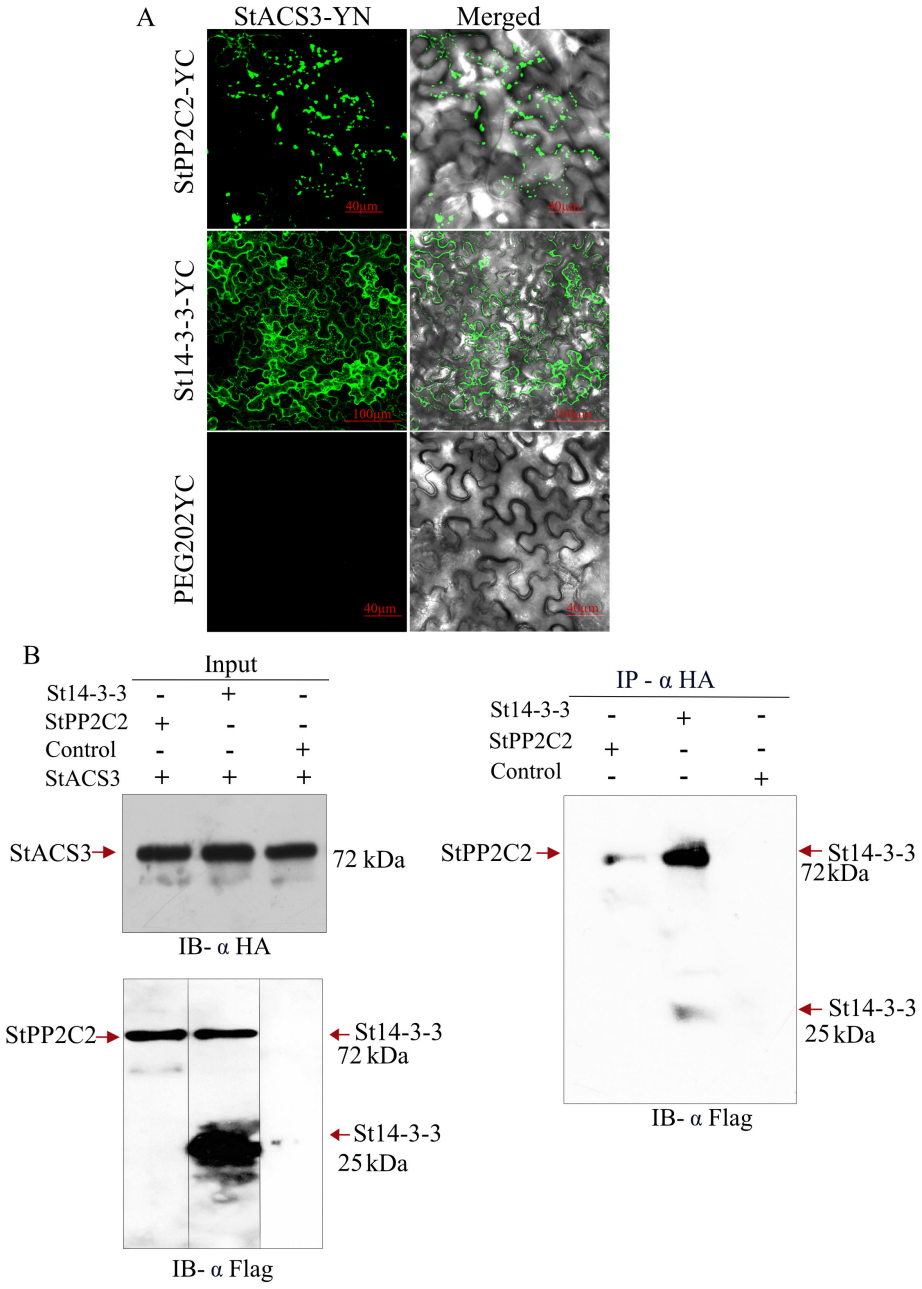
StACS3 interacts with StPP2C2 and St14-3-3 proteins *in vivo*. **(A)** BiFC assay between StACS3 and StPP2C2 or St14-3-3 proteins in *N. benthamiana*. StACS3 was fused with YFPn (YN) in the pEG201 vector, while StPP2C2 or St14-3-3 was fused with YFPc in the pEG202 vector. The use of a negative control with the empty YC vector yielded no fluorescence signal, thereby confirming the specificity of the observed interactions. Positive interactions were visualized as YFP signals using confocal microscopy, with Z-stack imaging performed at 10 µm intervals. **(B)** Co-immunoprecipitation of StACS3 with StPP2C2 and St14-3-3 in *N. benthamiana*. Crude leaf extracts from agroinfiltrated *N. benthamiana* expressing HA-tagged StACS3 and FLAG-tagged StPP2C2 or St14-3-3 were subjected to immunoprecipitation with an anti-HA antibody. FLAG-tagged StPP2C2 and St14-3-3 in the immunocomplex were detected using an anti-FLAG antibody. The left panels demonstrate input levels of StACS3, StPP2C2, and St14-3-3 proteins. Control samples comprise extracts from buffer-infiltrated leaves.

The interaction between StACS3 and StPP2C2 was investigated to elucidate the role of phosphatases in regulating ethylene biosynthesis enzymes. BiFC and Co-IP assays were used to determine whether this regulation occurs through direct interactions. BiFC analysis revealed a specific interaction between StACS3-YN and StPP2C2-YC, marked by bead-like fluorescence signals along the cellular membrane (**Figure 8A**, top), suggesting UPS-mediated degradation. St14-3-3 and StACS3 exhibited a more pronounced interaction, accompanied by a more uniform membrane signal (**Figure 8A**, middle panel), indicating that St14-3-3 may contribute to the stabilization of StACS3 and mitigate its degradation. The lack of fluorescence in the control containing the empty YC vector co-expressed with StACS3-YN confirmed the specificity of the observed interactions. These findings highlight the post-translational regulation of StACS3, with St14-3-3 enhancing its stability. To validate these interactions further, a Co-IP assay was performed. Using StACS3 as a bait, we successfully immunoprecipitated StPP2C2 and St14-3-3 from agroinfiltrated *N. benthamiana* leaf samples. The input samples verified the presence of the proteins used in the assay, as illustrated by immunoblotting (**Figure 8B**). StPP2C2 demonstrated strong interaction with StACS3, as evidenced by the detection of StPP2C2 in the Co-IP complex (**Figure 8B**). St14-3-3 exhibited both monomeric and heteromeric forms in input samples. However, after Co-IP, heteromers were more abundant, suggesting a stronger interaction between the heteromers of St14-3-3 and StACS3 (**Figure 8B**).

**Figure 9:**
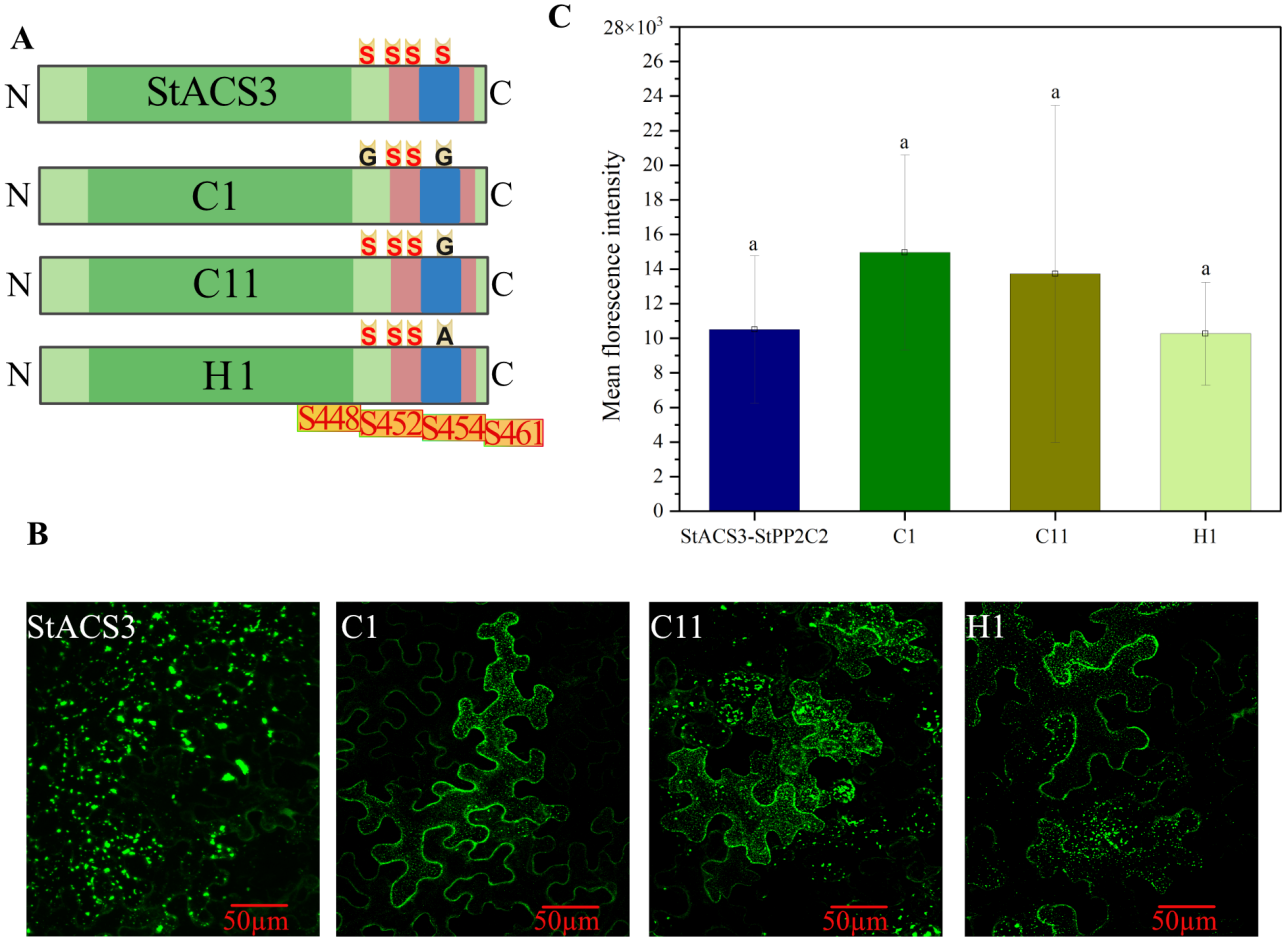
Interaction of StACS3 serine mutants with StPP2C2. **(A)** StACS3 serine mutants were generated by site-directed mutagenesis. C1 presents serine 461 (S461) and serine 448 (S448) substitutions with glycine (G). C11 has S461 to G and H1 presents S461 to alanine **(A)** substitution. All mutants were constructed in the pEG201-YC vector. **(B)** Confocal microscopy images showing BiFC-based interaction of full-length StACS3 and serine mutants (C1, C11, and H1) with StPP2C2 in *N. benthamiana* leaf epidermal cells. YFP fluorescence indicates protein–protein interaction. All panels are displayed with a uniform scale bar of 50 µm. All panels are presenting the maximum fluorescence intensity. **(C)** Quantification of mean fluorescence intensity of StACS3 and its mutants interacting with StPP2C2, calculated using ImageJ-based threshold analysis. Bar plots represent the average fluorescence intensity ± SD from multiple images (n = 9), generated using OriginPro software. Different letters indicate statistically significant differences based on one-way ANOVA followed by Tukey’s *post hoc* test (p < 0.05).

To investigate the role of specific serine residues within StACS3, phosphorylation site mutants (C1, C11, and H1) were generated, each targeting a conserved C-terminal serine residue, particularly serine 461 (S461), within the RLSF motif, which is potentially involved in post-translational regulation (**Figure 9A**). The serine mutants, C1 (S461G and S448G), C11 (S461G), and H1 (S461A), interacted significantly with StPP2C2. The StACS3-StPP2C2 complex was predominantly localized to the membrane and cytoplasm in a punctate form. However, the C1 mutant exhibited a slight enhancement in protein stability, as evidenced by reduced proteolysis and decreased punctate membrane localization (**Figure 9B**). Quantitative analysis of YFP fluorescence intensity revealed no significant difference in overall mutant protein accumulation compared to wild-type StACS3 (**Figure 9C**). Nevertheless, punctate-like structures were notably reduced in all mutants, with C1 displaying the highest degree of stabilization, suggesting that these mutations influence protein localization and turnover, likely by interfering with phosphorylation-dependent alterations in protein stability rather than affecting total protein levels.

These findings, supported by the BiFC data, underscore the critical roles of both St14-3-3 and StPP2C2 in regulating the stability and activity of StACS3. St14-3-3 enhances StACS3 stability by inhibiting degradation processes, whereas StPP2C2 appears to modulate its turnover, highlighting its collaborative regulatory mechanisms in plant physiology.

## Discussion

Globally, crop productivity is challenged by drought, particularly in potatoes, which are known to reduce plant growth, shorten the growth cycle, and limit tuber size (Obidiegwu et al., 2015; Dahal et al., 2019). Drought enhances ethylene production by increasing ACC-, a precursor synthesized through ACS accumulation (Rodrigues et al., 2014; Pattyn et al., 2021; Khan et al., 2024). Mechanistically, type II ACS proteins regulate ethylene biosynthesis via C-terminal Ser/Thr phosphorylation, influencing protein viability and ethylene production (Lee et al., 2017; Li et al., 2022).

In this study, we provided several lines of evidence that potato StACS3 regulates stress-induced ethylene production through interaction and modulation by StPP2C2 and St14-3-3 proteins, contributing to a negative regulatory effect on drought stress tolerance.

### StACS3 transcript upregulation and genetic modulation highlight its biological roles

We identified 16 full-length StACS gene sequences in the potato genome, including StACS3, a type II ACS isoform that contains conserved CDPK phosphorylation sites and an ETO1-binding domain. Phylogenetic analysis revealed high homology between StACS3 and type II ACS proteins from *A. thaliana* and tomato (**Figure 1**), highlighting its evolutionary conservation and potential role in stress responses (Yamagami et al., 2003; Kamiyoshihara et al., 2010).

Our results demonstrate that drought stress significantly increases type II StACS3 transcript accumulation (**Figure 2A**, **Supplementary Figure 1A**), which was further supported by elevated GUS reporter activity driven by the StACS3 promoter under PEG-induced stress conditions (**Figures 2C, D**). StACS3 promoter analysis revealed the presence of multiple stress-responsive cis-elements, including ABRE, MYC, MYB, ERF, W-box, and DOF motifs, consistent with regulatory elements as reported in other plant species (Plesch et al., 2001; Song et al., 2024). This study further aligns with prior findings showing that drought and hormonal signaling induce the expression of ACS genes such as AtACS4, AtACS5, and AtACS6, leading to modulated ethylene biosynthesis (Wang et al., 2005; Nemhauser et al., 2006).

Our tissue-specific transcript analysis revealed that StACS3 is highly expressed in branches, with progressively reduced expression in older leaves, stems, upper parts of primary roots, and flowers (**Figure 2B**). The StACS3 promoter-driven GUS assay further confirmed the higher GUS activity in apical meristems, hypocotyls, anthers, and vascular tissues, emphasizing its diverse roles in plant development (**Figure 2C**). Furthermore, in transgenic *A. thaliana*, StACS3-YFP localized predominantly in the root elongation and transition zones, consistent with the observed GUS expression pattern (**Figure 5G**). These findings highlight StACS3’s functional involvement in regions of high metabolic activity and its role in ethylene-mediated growth and stress response.

Ethylene overproduction in plants leads to phenotypic anomalies, including leaf epinasty, accelerated senescence, inhibited root growth, abnormal flowering, and fruit ripening (Wang et al., 2002; Iqbal et al., 2017). To further investigate the functional role of StACS3, transgenic *A. thaliana* plants expressing StACS3-YFP were examined. Heterologous expression of StACS3 conferred a stress-sensitive phenotype, mirroring the effects of ethylene overproduction. This phenotype is characterized by reduced plant height, increased sterile siliques, suppressed root growth, and accelerated leaf senescence (**Figure 5**), thereby elucidating StACS3’s role in mediating stress susceptibility through its contribution to ethylene production (Luo et al., 2014; Arraes et al., 2015; Booker and DeLong, 2015; Gamalero et al., 2023). While *A. thaliana* was selected for its genetic tractability and well-characterized ethylene pathway, the observed phenotypic manifestations may only partially align with those observed in potato. In particular, the role of StACS3 in tuber development under drought stress requires further investigation. In our study, transient silencing of StACS3 in Desiree using a ViGS construct designed to target the 5′ UTR, and evaluated for specificity against StACS10/12, significantly enhanced plant survival under drought conditions (**Figure 3A**), strongly implicating StACS3 in ethylene-mediated drought responses in potato (**Figures 4A, B**). While these results suggest that StACS3 silencing improves drought tolerance, potential off-target effects on closely related genes still cannot be denied. Therefore, generating stable overexpression or CRISPR-Cas knockout lines will be essential to assign this function to StACS3 definitively and to evaluate its impact on key agronomic traits, such as tuber yield and quality.

Interestingly, these results are consistent with previous studies on cereals and other crops, which have demonstrated the involvement of ACS family genes in regulating ethylene production during abiotic stress (Dong et al., 2011; Habben et al., 2014; Li et al., 2022). For, a drought study of maize seedlings showed an inverse relationship between endogenous ethylene levels and root elongation (Alarcon et al., 2009). Similarly, knockdown of ACS genes in maize has been documented to reduce ethylene emissions, which in turn mitigates drought-induced senescence in older leaves and enhances photosynthetic activity (Young et al., 2004). Interestingly, reduced ethylene production in StPP2C2-silenced plants suggested a post-translational regulatory mechanism. This paradox may be explained by the fact that StPP2C2 functions as a phosphatase that activates StACS3 through dephosphorylation. Its absence could lead to hyperphosphorylation and static protein expression, but altered enzymatic activity of StACS3, a mechanism consistent with the regulatory models established for AtACS6 and LeACS2 (Kamiyoshihara et al., 2010; Skottke et al., 2011). Although the direct enzymatic activity was not assessed in this study, the possibility of elevated StACS3 protein levels in StPP2C2-silenced plants warrants further investigation. Moreover, it is plausible that StPP2C2 has multiple targets among ACS isozymes, which aligns with the findings in *A. thaliana*, where type-specific phosphatases, such as PP2A, differentially modulate ACS6 (type I) instability while stabilizing ACS5 (type II) (Ludwikow et al., 2014; Skottke et al., 2011). Therefore, this multifaceted regulation of various ACS isozymes by StPP2C2 could be a key factor contributing to the decreased ethylene levels observed in StPP2C2-silenced plants. However, this potential multi-target regulation by StPP2C2 requires further investigation. In addition to the direct regulation of ACS isozymes, enhanced ABA signaling upon StPP2C2-silencing may antagonize ethylene biosynthesis (Cheng et al., 2009).

Collectively, this study contributes to the growing body of research (Young et al., 2004; Habben et al., 2014; Zhang et al., 2020b) linking the ACS gene family to both abiotic stress responses and plant development, positioning StACS3 as a key regulator in the drought tolerance-ethylene synthesis trade-offs.

### StACS3 silencing modulates ROS homeostasis and stress phenotype

The reduction in reactive oxygen species (ROS) accumulation in StACS3-silenced plants under drought conditions elucidates the role of StACS3 in maintaining ROS homeostasis (**Figures 4C–E**, **5H**), which is essential for metabolic adjustments that enhance drought resilience (Wang and Huang, 2019). Decreased ROS, particularly hydrogen peroxide (H_2_O_2_), in silenced plants under drought conditions suggests an improved oxidative balance, as evidenced by the reduced ion leakage from leaf tissues after 14 days of drought (**Figure 4C**). Conversely, overexpression of StACS3 resulted in elevated ROS levels and increased sensitivity to drought (**Figure 5H**), emphasizing StACS3’s role in oxidative stress response. These findings are consistent with those reported for rice, where drought-tolerant cultivars exhibited reduced oxidative damage and efficient osmotic adjustments (Wang et al., 2019). The significant reduction in ROS levels in StACS3-silenced plants highlights the potential of modulating ethylene and ROS pathways to improve plant adaptation to abiotic stress, corroborating previous findings on stress-tolerance mechanisms (Steffens, 2014; Wang and Huang, 2019).

### StACS3 protein expression and distribution under proteasome inhibition and stress conditions

The expression and localization of the StACS3 protein were significantly enhanced under drought stress and MG132 treatment (**Figure 6**). ACS proteins are critical regulators of ethylene biosynthesis, and their expression and stability are tightly regulated by phosphatase activity and ubiquitination to maintain cellular homeostasis (Tsuchisaka et al., 2009; Lee et al., 2017, 2021). Although ethylene production typically remains low under normal growth conditions, its levels increase substantially during stress responses or specific developmental stages (Chen et al., 2021).

Our results indicated that MG132 treatment led to a significant increase in StACS3 protein expression and a transition in protein localization from a punctate pattern to a more stable, membrane-bound form (**Figure 6**). Enhanced StACS3 localization has been observed in guard cells, suggesting that proteasome inhibition promotes stabilization and redistribution of StACS3 from a degraded state to a functionally distinct form (Wang et al., 2004; Tan and Xue, 2014). Furthermore, PEG treatment induced a shift in StACS3 localization to cell membranes, particularly to guard cells (**Figure 6**). This alteration supports the hypothesis that ACS proteins, including StACS3, contribute to stomatal function and cellular adaptation to stress (Tsuchisaka et al., 2009; Seo and Yoon, 2019). Consistently, transgenic StACS3-YFP showed its primary localization in the stomata (**Figure 4**, **Supplementary Figure 2**), indicating a potential functional role for StACS3 in these structures (Tanaka et al., 2005; Chen et al., 2013, 2021).

Although this study provides evidence for StACS3’s involvement in stress-induced protein stabilization in stomata, the precise mechanisms underlying its regulation of stomatal function remain largely uncharacterized. Previous studies have indicated that the coordinated expression of ACS family members in guard cells suggests their broader role in stomatal physiology (Tsuchisaka and Theologis, 2004; Tsuchisaka et al., 2009; Hasan et al., 2024). These findings emphasize the need for further investigations to elucidate the role of StACS3 in stomatal regulation and plant stress responses.

### PP2C2-silencing restores the stability and protein expression of StACS3

The C-terminus of the StACS3 protein contains specific phosphorylation sites that render it susceptible to targeted ubiquitination, a process mediated by ETO1/EOL proteins, that marks ACS enzymes for degradation (**Figure 1C**) (Yoshida et al., 2005, 2006; Christians et al., 2009; Lee et al., 2021). Conversely, dephosphorylation by protein phosphatases has also been reported to promote ETO1-mediated degradation, suggesting that the phosphorylation status is critical for determining ACS protein stability (Skottke et al., 2011; Lee et al., 2021).

In potato, the PP2C gene family comprises 78 members, some of which function as negative regulators of abscisic acid (ABA) signaling under various environmental stress conditions (Wang et al., 2020). PP2C proteins influence the stress response of plants by interacting with SnRK2 kinases, either by inhibiting their phosphorylation or by directly dephosphorylating target proteins (Jung et al., 2020). This mechanism is essential for water stress responses, where ABA receptors (PYL/PYR) interact with PP2Cs and influence downstream transcriptional regulation (Umezawa et al., 2009; Geiger et al., 2010; Fuchs et al., 2013).

Previous studies have revealed that drought-sensitive potato cultivars harbor additional alleles of StPP2C2. In contrast, drought-tolerant cultivars carry a single copy, suggesting a possible role for StPP2C2 in abiotic stress signaling and adaptation (Schumacher et al., 2021).

In this study, ectopic transient expression of StACS3-YFP in *N. benthamiana* leaves with silenced StPP2C2 resulted in significantly enhanced and prolonged fluorescence signals, detectable from 16 hours and persisting up to 7–10 days post-infiltration (dpi) (**Figures 7B, C**). Notably, the fluorescence intensity peaked around 64 hours post-infiltration (hpi), exceeding the typical transient expression kinetics in *N. benthamiana*, where protein accumulation usually peaks between 36 and 48 hpi and declines thereafter due to natural turnover (Voinnet et al., 2003). This prolonged signal suggests that StPP2C2 facilitates post-translational destabilization of StACS3, contributing to its degradation under normal conditions. We note that at 80 hrs, the overall signal intensity was markedly decreased (**Figure 7C**). However, a faint membrane-localized pool remained detectable under maximum-intensity projections (**Figure 7B**), potentially representing a stable fraction less accessible to degradation machinery (Eilertsen et al., 2024). These interpretations are further supported by an *in vitro* degradation assay, in which co-expression of StPP2C2 led to reduced StACS3-YFP levels, consistent with a role in modulating the protein’s phosphorylation status to promote proteasomal degradation (**Figure 7D**).Western blot analysis confirmed the increased accumulation of StACS3 in StPP2C2-silenced tissues (**Figure 7A**). Moreover, increasing concentrations of StPP2C2 resulted in altered StACS3 mobility and the appearance of degradation-associated smears and ladder-like bands (**Figure 7D**), indicative of post-translational modifications, such as ubiquitination.

The absence of a discrete, stabilized StACS3 band in lane 2 (1:0.1 ratio), which instead shows a diffuse pattern, may reflect its basal phosphorylation state and the initial phase of targeted degradation already occurring at this low StPP2C2 concentration. Although a marked reduction in total StACS3 protein was not immediately evident, the presence of degradation intermediates suggests that StPP2C2 may act to prime StACS3 for gradual proteasomal degradation rather than initiating rapid turnover. These findings align with those of previous studies in *A. thaliana*, where PP2Cs facilitated proteasome-mediated degradation of type I/III ACS proteins by reducing their phosphorylation (Cotelle and Leonhardt, 2016; Ormancey et al., 2017; Marczak et al., 2020). Interestingly, our findings diverge from reports on type II ACS proteins in *A. thaliana*, where inhibition of phosphatase activity by cantharidin destabilized ACS5, a phenotype also observed in the rcn1 phosphatase mutant (Skottke et al., 2011; Park et al., 2021). These results suggested that phosphatase inhibition leads to enhanced phosphorylation and accelerated degradation (Lyzenga and Stone, 2012). In contrast, our data show that silencing StPP2C2 in potato stabilizes StACS3, likely by altering its phosphorylation dynamics, pointing to a species-specific regulatory mechanism.

Further validation through cycloheximide chase assays and transgenic lines expressing phospho-mutant variants of StACS3 would be valuable to clarify the precise role of StPP2C2 in modulating StACS3 turnover. Collectively, these findings emphasize the value of species-specific studies to uncover distinct regulatory modules and inform targeted strategies for improving stress resilience in crops.

### Complex modulation of StACS3 stability through differential interactions with StPP2C2 and St14-3-3

The regulation of ACS proteins involves complex interactions with type-specific phosphatases and 14-3-3 proteins, each exerting distinct regulatory roles. These phosphatases, such as PP2A, modulate ACS stability in a type-dependent manner, destabilizing type I ACS6 while stabilizing type II ACS5 (Skottke et al., 2011; Ludwikow et al., 2014). Conversely, 14-3-3 proteins are crucial for stabilizing ACS proteins by binding to phosphorylated targets, including ETO1/EOLs, components of the Cullin-3 E3 ubiquitin ligase complex. Yoon and Kieber (2013) proposed that this binding disrupts E3 ligase function, thereby preventing proteasome-mediated degradation of ACS proteins.

Interestingly, our BiFC experiment revealed contrasting interaction dynamics between StACS3 and its two regulators, StPP2C2 and St14-3-3. A transient interaction, visualized as punctuate structures, was observed between StPP2C2 and StACS3, persisting for only 2–3 dpi. In contrast, the interaction between St14-3-3 and StACS3 was more stable, lasting 7–10 dpi (**Figure 8A**). The transient nature of the StPP2C2-StACS3 interaction suggests that StPP2C2 promotes StACS3 dephosphorylation, facilitating its degradation. This is corroborated by our protein degradation assay (**Figure 7D**) and aligns with previous studies (Marczak et al., 2020). Conversely, the stable interaction between St14-3-3 and StACS3 implies that St14-3-3 stabilizes StACS3, potentially by shielding it from proteasomal degradation. These differential regulatory roles were further validated through Co-IP assays. StACS3 was found to associate with both monomeric and heteromeric forms of St14-3-3, consistent with the known function of 14-3-3 proteins in stabilizing phosphorylated substrates (Yoon and Kieber, 2013). In contrast, the interaction with StPP2C2 suggests a regulatory mechanism involving targeted dephosphorylation of StACS3 (**Figure 8B**).

Localization studies of StACS3-YFP in guard cells support its role in stomatal regulation, as proposed in earlier studies (Tanaka et al., 2005; Chen et al., 2013; Hasan et al., 2024). This observation aligns with the broader understanding that 14-3-3 proteins are generally enriched in the stomata, where they play key regulatory roles. For example, Hv14-3-3A mediates stomatal closure and enhances drought tolerance in barley (Jiang et al., 2023). This suggests that StACS3 may function in conjunction with or parallel to 14-3-3 proteins to regulate stomatal responses under stress conditions.

Similar to wild-type StACS3, all serine-mutated variants exhibited a non-steady proteolytic signal, indicating that phosphorylation loss at these residues does not entirely abolish the protein’s dynamic turnover (**Figures 9A, B**). However, confocal microscopy revealed that the serine-to-Gly/Ala substitutions conferred partial stabilization of the mutants compared to the wild-type StACS3-StPP2C2 complex (**Figure 9**). Among the variants, the C1 mutant targeting S461 and S448 showed the most pronounced stabilization effect. These findings suggest that phosphorylation of these specific sites plays a pivotal role in regulating StACS3 protein stability.

Phosphorylation often functions as a molecular switch or rheostat, modulating protein activity, localization, and degradation. This dual regulatory capacity enables the precise control of protein turnover (Landry et al., 2014). Although our quantitative analysis did not reveal statistically significant differences in overall fluorescence intensity between the mutants and the wild-type control, the spatial distribution of fluorescence was notably altered (**Figures 9B, C**). These results are consistent with previous findings in *A. thaliana*, where mutations disrupting CDPK-mediated phosphorylation sites in ACS6, a type I ACS, resulted in increased protein stability and enhanced ethylene production without affecting enzymatic activity (Joo et al., 2008; Kamiyoshihara et al., 2010; Booker and DeLong, 2015; Lee et al., 2021). The conservation of regulatory mechanisms across plant species reinforces the importance of phosphorylation in ACS protein turnover. Combining StPP2C2 overexpression with MG132 treatment could further clarify the role of the proteasome; the dynamic nature of concurrent protein synthesis and degradation in the transient system presents a methodological challenge that underscores the need for complementary approaches. To address this, future studies employing cycloheximide chase assays in stable transgenic lines will be critical to determine StACS3 recovery from proteasomal inhibition under PP2C2 overexpression. Our data support a model in which reduced phosphorylation, either through site-directed mutagenesis or silencing of the phosphatase StPP2C2, leads to the stabilization of StACS3. This stabilization may, in turn, elevate ethylene biosynthesis under stress conditions such as drought. To substantiate this hypothesis and to delineate the molecular underpinnings of ethylene-mediated drought tolerance, further functional validation using stable transgenic potato lines expressing phospho-deficient StACS3 variants will be essential.

Collectively, these results provide novel insights into the post-translational regulation of type II ACS proteins in potato, distinguishing their regulatory mechanisms from those of *A. thaliana* ACS proteins. These findings lay the groundwork for future strategies aimed at manipulating ethylene biosynthesis to enhance the drought resilience of crop species.

**Figure 10:**
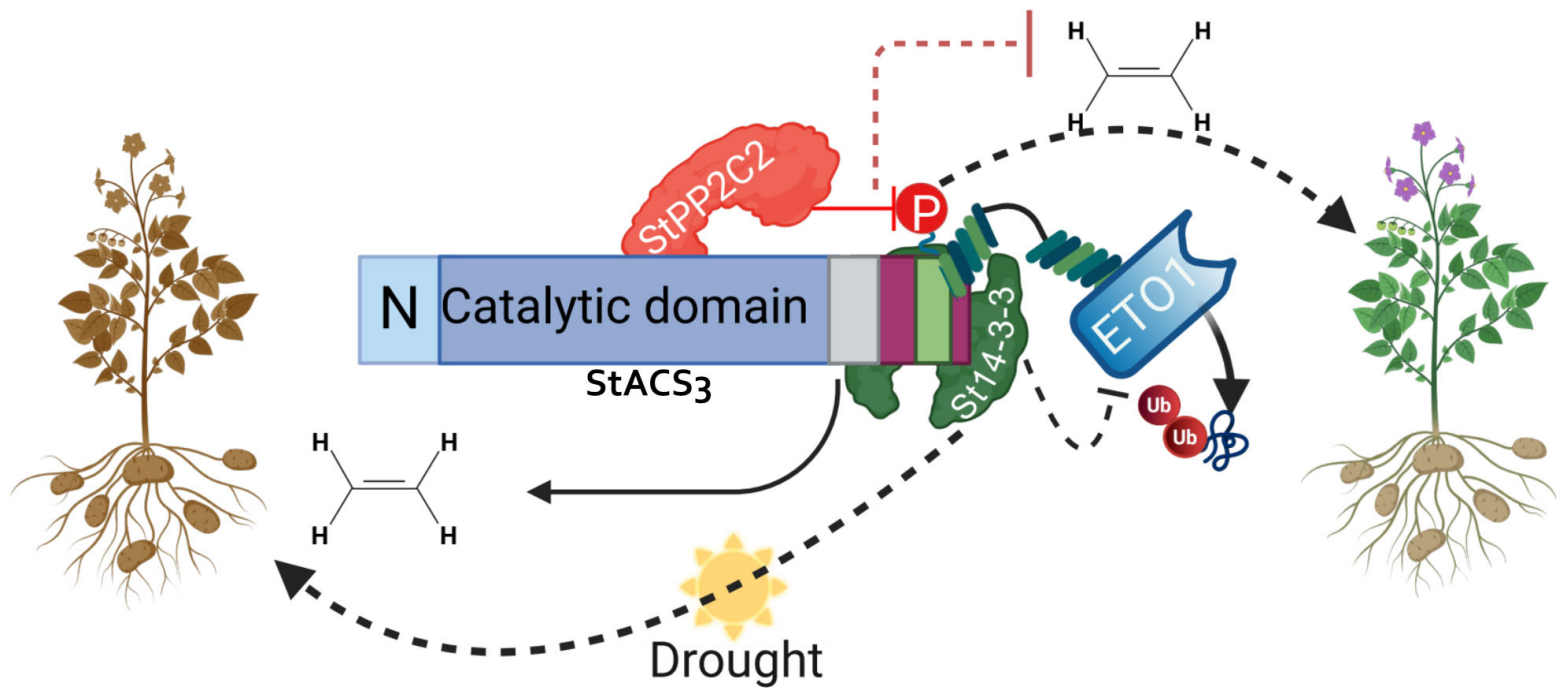
Model of StACS3 regulation during drought stress in potato. StACS3 is a Type II ACS protein that contains a conserved catalytic domain and C-terminal regulatory region with phosphorylation sites. Under drought stress, StACS3 is transcriptionally and post-translationally upregulated, contributing to an enhanced ethylene biosynthesis and drought adaptation. Phosphorylation of StACS3 promotes protein stabilization and facilitates its interaction with 14-3-3 proteins (St14-3-3), which shields StACS3 from degradation. In contrast, under controlled growth conditions, StPP2C2 dephosphorylates StACS3, potentially priming it for ubiquitination by ETO1 and subsequent degradation via the 26S proteasome.

In conclusion, our model (**Figure 10**) illustrates the regulatory framework of StACS3 in *S. tuberosum* under drought stress, highlighting key insights into its role in drought tolerance. Our data indicate that drought induces both transcriptional activation and post-translational stabilization of the type II ACS isozyme StACS3. Silencing of StACS3 reduces ethylene production and enhances drought tolerance, mimicking the stress-resilient phenotype observed in the cultivar Desiree. In contrast, overexpression of StACS3 results in pronounced developmental abnormalities, including impaired root elongation, reduced plant stature, accelerated leaf senescence, and elevated hydrogen peroxide (H_2_O_2_) accumulation, underscoring the importance of tight post-transcriptional and post-translational regulation of StACS3 during stress adaptation.

### Limitations and future perspectives

Our study establishes a compelling model for the post-translational regulation of StACS3 stability by StPP2C2 and St14-3-3; however, several key aspects of this mechanism demand further investigation. First, the direct impact of StPP2C2- or St14-3-3-binding on the enzymatic activity of StACS3 needs quantitative analysis. Second, delineating the precise spatiotemporal dynamics of these protein complexes within native potato cells, particularly in response to drought stress, will be crucial for understanding the regulatory mechanism *in vivo*. Finally, determining the broader regulatory scope of StPP2C2 and St14-3-3, specifically, whether they orchestrate a coordinated ethylene response by targeting multiple ACS isozymes, represents a key objective for future interactome studies.

To address these limitations and build upon our findings, we propose several future directions. The generation and phenotypic analysis of stable CRISPR-Cas knockout mutants for *StACS3* and *StPP2C2* in potato are essential to unequivocally confirm their physiological roles in drought tolerance and ethylene biosynthesis, thereby circumventing compensatory effects associated with transient silencing. These genetic resources would be invaluable for conducting detailed stomatal aperture assays and elucidating the intricate crosstalk between ABA and ethylene signaling pathways. Furthermore, employing advanced biophysical techniques such as Förster Resonance Energy Transfer (FRET) could reveal the real-time dynamics and stoichiometry of these interactions in living plant cells. Ultimately, a deeper mechanistic understanding of this regulatory node could facilitate developing drought-resilient potato cultivars through the precise engineering of ethylene synthesis and signaling pathways.

## Data Availability

The original contributions presented in the study are included in the article/**Supplementary Material**. Further inquiries can be directed to the corresponding author, Sadia Hamera, or, if necessary, to the co-corresponding authors.

## Supplementary material

Supplementary Figure 1 Transcript accumulation of StACS3 in potato varieties and histochemical staining of StACS3::GUS promoter-driven plants under stress conditions.

Supplementary Figure 2 Subcellular localization of StACS3 in transgenic *A. thaliana*.

Supplementary Figure 3 Phylogenetic analysis and subgrouping of ACC synthase (ACS) proteins from potato, tomato, and Arabidopsis.

Supplementary Table 1 List of cis-regulatory elements in StACS3 promoter sequence.

Supplementary Table 2 GUS expression in different organs of transgenic *A. thaliana* plants.

Supplementary Table 3 List of primers.

Supplementary Table 4 Accession numbers and identifiers of genes used in this study.

Supplementary Table 5 Statistical significance of pairwise comparisons for DAB assay and Electrolyte conductivity.

Supplementary Table 6 Potato ACS protein accession numbers (IDs) and their classification.

Supplementary File 1 Potato StACS protein sequences.
