# Supplementary figures and images for: StACS3-mediated drought stress adaptation in potato involves interactions with StPP2C2 and St14-3-3 proteins
Source: Front Plant Sci. 2025 Oct 30;16:1671817. doi: 10.3389/fpls.2025.1671817 (PMC12611960; doi:10.3389/fpls.2025.1671817)

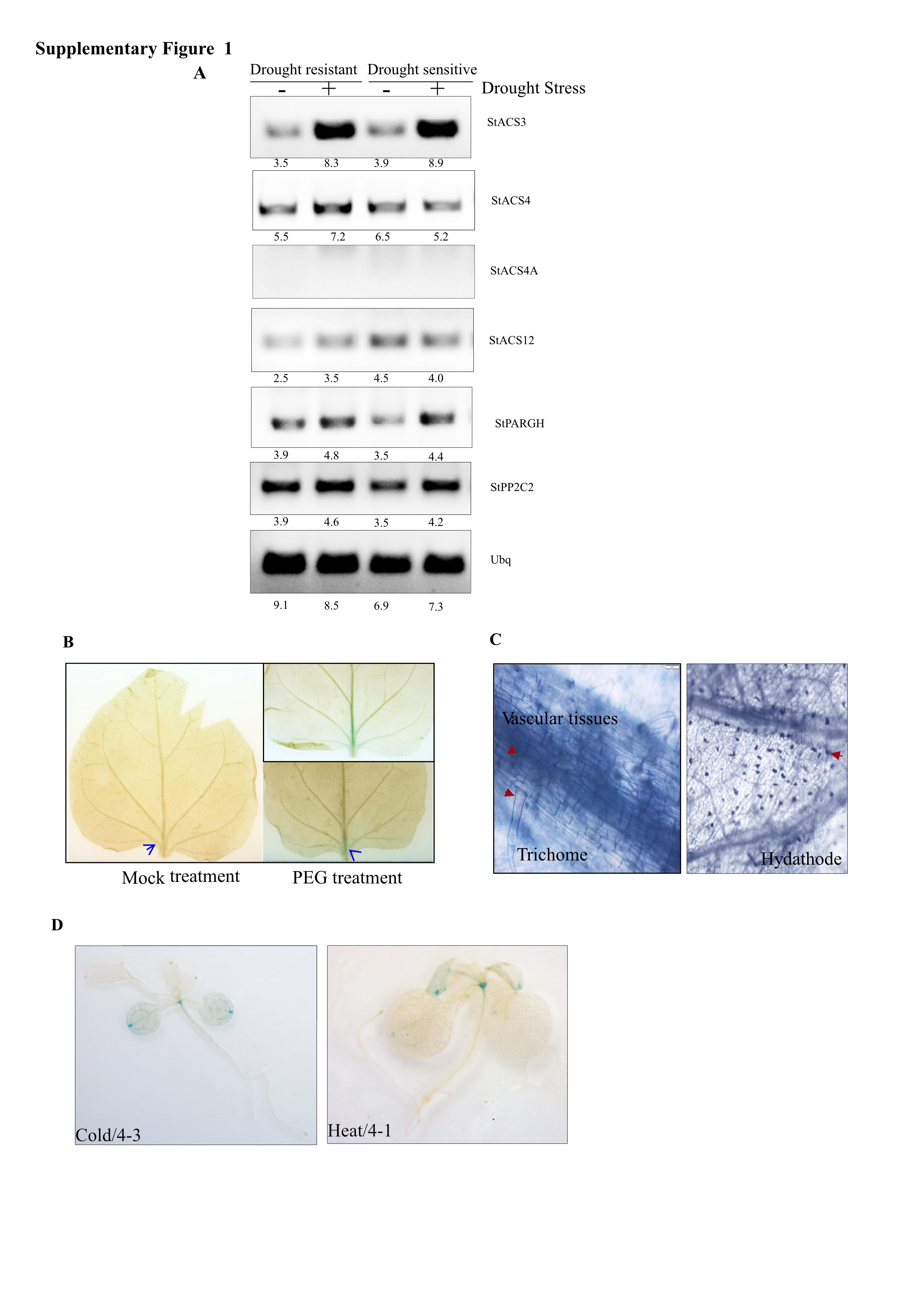

Supplement: Supplementary Figure 1 — Transcript accumulation of StACS3 in potato varieties and histochemical staining of StACS3::GUS promoter-driven plants under stress conditions. [file Image1.tiff]

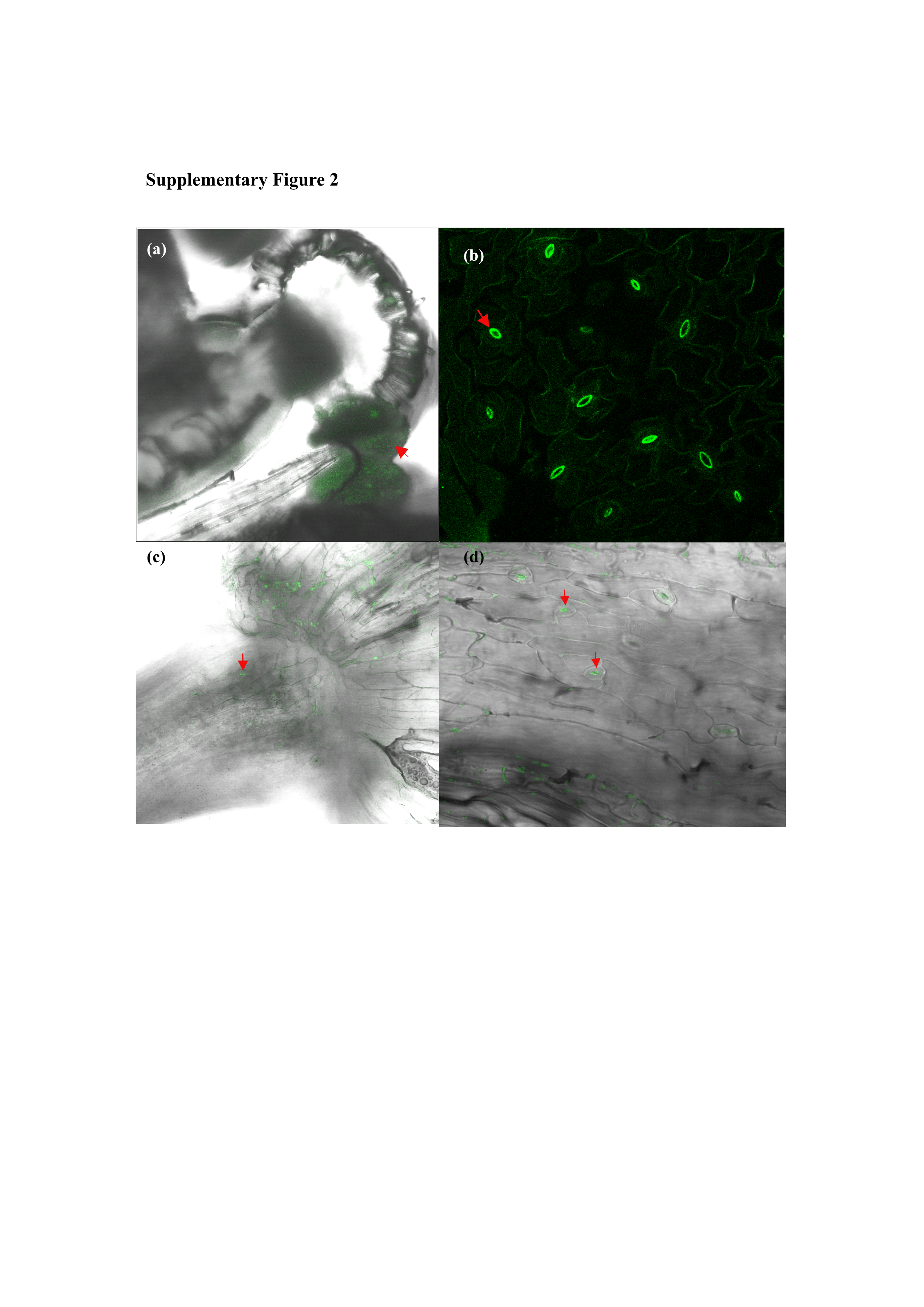

Supplement: Supplementary Figure 2 — Subcellular localization of StACS3 in transgenic A. thaliana. [file Image2.tiff]

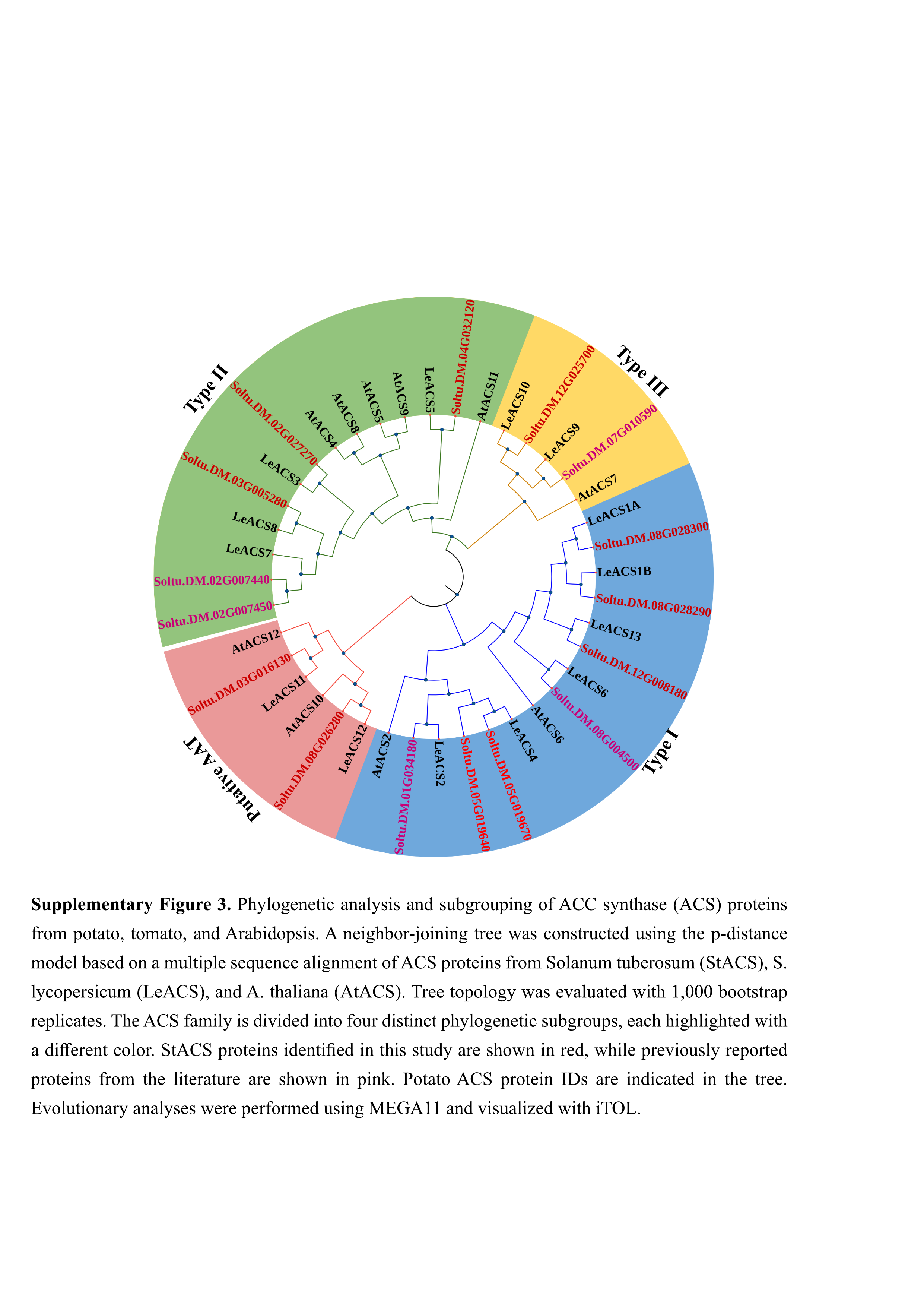

Supplement: Supplementary Figure 3 — Phylogenetic analysis and subgrouping of ACC synthase (ACS) proteins from potato, tomato, and Arabidopsis. [file Image3.tiff]
