# Supplementary material for: StACS3-mediated drought stress adaptation in potato involves interactions with StPP2C2 and St14-3-3 proteins
Source: Front Plant Sci. 2025 Oct 30;16:1671817. doi: 10.3389/fpls.2025.1671817 (PMC12611960; doi:10.3389/fpls.2025.1671817)
Supplement: Supplementary Table 1 — List of cis-regulatory elements in StACS3 promoter sequence. [file DataSheet1.pdf]

Supplementary Table 1: cis-regulatory elements in the StACS3 promoter sequence

Cis regulatory elements	Position(bp)	Sequence
MYC	-1534	CATGTG
W Box (WRKY38 binding site)	-105, -839	TTGAT/C
G Box	-801	CACGTG
ERF	-821	TGACY
ABRE	-1536, -803	GCCATGTC
Dof domain	-984	TAAAGSTKST1
