# Supplementary material for: StACS3-mediated drought stress adaptation in potato involves interactions with StPP2C2 and St14-3-3 proteins
Source: Front Plant Sci. 2025 Oct 30;16:1671817. doi: 10.3389/fpls.2025.1671817 (PMC12611960; doi:10.3389/fpls.2025.1671817)
Supplement: Supplementary Table 2 — GUS expression in different organs of transgenic A. thaliana plants. [file DataSheet2.pdf]

Supplementary Table 2. StACS3 promoter driven GUS expression in different tissues of transgenic *A. thaliana*

**Plant organ**	**-1.2 MPa***	**-0.5 MPa***
Cotyledon	yes	yes
Primary leaves	yes	yes
Apical meristem	yes	yes
Hypocotyl	yes	yes
Embryonic root vasculature	yes	yes
Leaf hydathodes	yes	yes
Root tip	no	no
Root meristem	no	no

*MPa- Megapascal
