# Supplementary material for: StACS3-mediated drought stress adaptation in potato involves interactions with StPP2C2 and St14-3-3 proteins
Source: Front Plant Sci. 2025 Oct 30;16:1671817. doi: 10.3389/fpls.2025.1671817 (PMC12611960; doi:10.3389/fpls.2025.1671817)
Supplement: Supplementary Table 3 — List of primers. [file DataSheet3.pdf]

Supplementary Table 3

Primer names	Sequences (5' to 3')
**Relative gene expression**	
ACS3for2	GCTCAAGTTCAAACGGATTCA
ACS3rev2	AGATCTCGTCACTGATGAGG
PP2C Arev	AGTTTACCAGGCTCCTGTTCC
PP2C Bfor	TCAACGGAAGAGATGCAGCTG
F1 PARG1	CACCCGGAAATCTCAAAATGGCAAA
R1 PARG1	TCATAGAGGTGCCAAACAT
F-St Ubq	CTGGCAAGACCATAACTCTCGA
R-St Ubq	CTTTCCAGCAAAGATCAGCCTT
F- St ACS4	ATGATGGAACGGTGGATA
R- St ACS4	TGAGGCAGGAATAGGAGA
F- St ACS4A	CGATAGGGATTTATGTTG
R- St ACS4A	AAGGTCGTTAGTGAAGGT
F StACS12	TTTCTTGTTCCCTCACCA
R StACS12	CGTACTTTAAGGCCACGT
F St ACS10	ACCACAAGCCTTAGAATC
R St ACS10	TGCTGAGCCAGAGTAGAT
F1 St.PDSRT	CTACTGGAGGCAAGGGATG
R1 St.PDS RT	TTAAAGGAGCGGGTAAAGC
**Promoter sequence**	
F-pACS3 1.8kb	ACtctagaTTCTCCGTAGTCTAGTGGTC
R-pACS3 1.8kb	acGGATCTagatct AATCAACTTGGAGATTGTAG
**ViGs Sequences**	
1F-StACS3 vigs1	cacc CTGGCACATTCCAGTCGAT
1R-StACS3 vigs1	TTGAAAGCAGGAAGACCAT
F-StACS3 TRV2-G	caccTAACGAAGCCTGGCGAGTT
2R-StACS3 vigsLIC rev	TATCCTGGATTCGATAGAG
2R-PP2C ViGS2	AGTTCAGGCTGTTTGTTC
2F-PP2C ViGS2	cacc TAAATGAGTCGCATGGTT
F1-StPDS	CTGACGAACTTTCAATGCAGT
R-StPDS	ACCATATATGTACATTTATAA
**mRNA cloning**	
F-ACS3 BamHI	ACTGGATCCATGAAGCTATTGTCAGAGAAA
R-ACS3 XhoI	AGACTCGAGGAATGTTCACAATTGCAGGAC
**BiFC Primers**	
PEG201YN ACS3 fw	ATGAAGCTATTGTCAGAGAAAG
PEG201YN ACS3 rev	TCGTTCTCTTTGACGGTCATT
PEG202Yc 14-3 Fw	ATGGCGTCGCCACGCGAGGAA
PEG202Yc 14-3 Rw	TTCATTATTATCTGGTTTAG
PEG202Yc PP2c Fw	GGATCCATGCATGCACAATCAATAACT
PEG202Yc PP2c Rev	GGATCCTTGGTATGTGGATGCTTTTGAGGT
ACS3 + pEG104_fwd	ATGAAGCTATTGTCAGAGAAAG
ACS3 + pEG104_rev	TATCGTTCTCTTTGACGGTC

**RT-qPCR Primers**	
Fw Actin X55749	GCTTCCCGATGGTCAAGTCA (Tang *et al*. 2017)
Rev Actin X55749	GGATTCCAGCTGCTTCCATTC (Tang *et al*. 2017)
Fw StACS3	GCTCAAGTTCAAACGGATTCA
Rev StACS3	AGATCTCGTCACTGATGAGG
Fw StACS1B	ACACTCATATTTTGCCTTGCTG
Rev StACS1B	CCGGTTCTCCATTTTAGGTCTC
StPP2C2-F	CTGACCGTCTGGGTTTTTGT (Wang *et al*. 2020)
StPP2C2-R	CTGACCGTCTGGGTTTTTGT (Wang *et al*. 2020)
EF1a-For	GATGGTCAGACCCGTGAACA (Tang *et al.* 2017)
EF1a-Rev	CCTTGGAGTACTTCGGGGTG (Tang *et al*. 2017)
**Mutagenesis primers**	
ACS3 rev S454G, S452G	CACTTGCCGAATCCCTTCTTCTTTCCATTAGCGTTAGA
ACS3 rev S454, S452	CACTTGGAGAATGACTTCTTCTTTGAATTAGCGTTAGA
ACS3 rev S461	CGGTCATTGAACGATAGTCGAAAAACCCACTTGGAGAATGA
ACS3 rev S461Ala	CCGTCATTGAACGCTAGTCGAAAAACCCACTTGGAGAATGA
ACS3 rev S461G	CGGTCATTGAACCCTAGTCGAAAAACCCACTTGGAGAATGA

References:

Tang X, Zhang N, Si H, Calderón-Urrea A. (2017) Selection and validation of reference genes for RT-qPCR analysis in potato under abiotic stress. Plant Methods. **16**;13:85.

Wang YF, Liao YQ, Wang YP, Yang JW, Zhang N, Si HJ (2020) Genome-wide identification and expression analysis of StPP2C gene family in response to multiple stresses in potato (*Solanum tuberosum* L.). Journal of Integrative Agriculture **19** (6):1609-1624.
