# Supplementary material for: StACS3-mediated drought stress adaptation in potato involves interactions with StPP2C2 and St14-3-3 proteins
Source: Front Plant Sci. 2025 Oct 30;16:1671817. doi: 10.3389/fpls.2025.1671817 (PMC12611960; doi:10.3389/fpls.2025.1671817)
Supplement: Supplementary Table 4 — Accession numbers and identifiers of genes used in this study. [file DataSheet4.pdf]

Supplementary Table 4: Accession numbers and identifiers of genes used in this study

Solanum tuberosum		*Solanum lycopersicum*		*Arabidopsis thaliana*	
**Gene Name**	**Gene ID v6.1**	**Gene Name**	**Gene ID**	**Gene Name**	**Gene ID**
**StACS3**	Soltu.DM.02G027270.1	**LeACS3**	Solyc02g091990	**AtACS4**	AT2G22810.1
**StACS1A**	Soltu.DM.02G007450.1	**LeACS5**	Solyc04g077410	**AtACS5**	AT5G65800.1
**StACS1B**	Soltu.DM.02G007440.1	**LeACS7**	Solyc02g063540	**AtACS8**	AT4G37770.1
**StACS8**	Soltu.DM.03G005280.1	**LeACS8**	Solyc03g043890	**AtACS9**	AT3G49700.1
**StACS15**	Soltu.DM.04G032120.1			**AtACS11**	AT4G08040.1
**StACS4**	Soltu.DM.01G034180.1	**LeACS1A**	Solyc08g081550	**AtACS2**	AT1G01480.1
**StACS4A**	Soltu.DM.05G019640.1	**LeACS1B**	Solyc08g081540	**AtACS6**	AT4G11280.1
**StACS4B**	Soltu.DM.05G019670.1	**LeACS2**	Solyc01g095080		
**StACS5**	Soltu.DM.08G004500.1	**LeACS4**	Solyc05g050010		
**StACS13**	Soltu.DM.12G008180.1	**LeACS6**	Solyc08g008100		
**StACS14A**	Soltu.DM.08G028300.1	**LeACS13**	Solyc12g056180		
**StACS14B**	Soltu.DM.08G028290.1				
**StACS10**	Soltu.DM.12G025700.1	**LeACS10**	Solyc12g008740	**AtACS7**	AT4G26200.1
**StACS2**	Soltu.DM.07G010590.1	**LeACS9**	Solyc07g026900		
**StACS11**	Soltu.DM.03G016130.1	**LeACS11**	Solyc03g007070	**AtACS10**	AT1G62960.1
**StACS12**	Soltu.DM.08G026280.1	**LeACS12**	Solyc08g079750	**AtACS12**	AT5G51690.1

**StPP2C2**	Soltu.DM.01G021880.1				
**StPDS**	Soltu.DM.03G037550.1				
**St14-3-3**	Soltu.DM.11G003450.1				
