# Supplementary material for: StACS3-mediated drought stress adaptation in potato involves interactions with StPP2C2 and St14-3-3 proteins
Source: Front Plant Sci. 2025 Oct 30;16:1671817. doi: 10.3389/fpls.2025.1671817 (PMC12611960; doi:10.3389/fpls.2025.1671817)
Supplement: Supplementary Table 5 — Statistical significance of pairwise comparisons for DAB assay and Electrolyte conductivity. [file DataSheet5.pdf]

Supplementary Table 5

A. Statistical significance of pairwise comparisons for electrolyte leakage

Comparison	P-Value	Significance
**pds vs. Control**	0.36818	Not Significant (ns)
**acs3 vs. Control**	< 0.0001	****
**acs3 vs. pds**	< 0.0001	****

B. Statistical significance of pairwise comparisons for DAB staining intensity

Comparison	P-Value	Significance
**EV-2wad vs. Control**	< 0.0001	****
**acs3-2wad vs. Control**	0.09921	Not Significant (ns)
**acs3-2wad vs. EV-2wad**	< 0.0001	****
