# Supplementary material for: StACS3-mediated drought stress adaptation in potato involves interactions with StPP2C2 and St14-3-3 proteins
Source: Front Plant Sci. 2025 Oct 30;16:1671817. doi: 10.3389/fpls.2025.1671817 (PMC12611960; doi:10.3389/fpls.2025.1671817)
Supplement: Supplementary Table 6 — Potato ACS protein accession numbers (IDs) and their classification. [file DataSheet6.pdf]

Supplementary Table 6: Potato Protein accession numbers (IDs)

Protein Type	Protein ID (Soltu, PGSC v6.1)	Protein Designations	Previous Gene names	Accession no.	Reference(s)
TypeI	Soltu.DM.05G019640.1	StACS4A			
TypeI	Soltu.DM.05G019670.1	StACS4B			
TypeI	Soltu.DM.01G034180.1	StACS4	StACS4	L20634	Schlagnhaufer et al. (1997)
TypeI	Soltu.DM.08G004500.1	StACS5	StACS5	U70842	Schlagnhaufer et al. (1997)
TypeI	Soltu.DM.12G008180.1	StACS13			
TypeI	Soltu.DM.08G028300.1	StACS14A			
TypeI	Soltu.DM.08G028290.1	StACS14B			
TypeIII	Soltu.DM.12G025700.1	StACS10			
TypeIII	Soltu.DM.07G010590.1	StACS2	StACS2	Z27235	Destefano-Beltran et al. (1995)
TypeII	Soltu.DM.04G032120.1	StACS15			
TypeII	Soltu.DM.02G027270.1	StACS3			
TypeII	Soltu.DM.02G007450.1	StACS1A	StACS1A	Z27233	Destefano-Beltran et al. (1995)
TypeII	Soltu.DM.03G005280.1	StACS8			
TypeII	Soltu.DM.02G007440.1	StACS1B	StACS1B	Z27234	Destefano-Beltran et al. (1995)
AAT	Soltu.DM.03G016130.1	StACS11			
AAT	Soltu.DM.08G026280.1	StACS12			
