# Supplementary material for: StACS3-mediated drought stress adaptation in potato involves interactions with StPP2C2 and St14-3-3 proteins
Source: Front Plant Sci. 2025 Oct 30;16:1671817. doi: 10.3389/fpls.2025.1671817 (PMC12611960; doi:10.3389/fpls.2025.1671817)
Supplement: Supplementary File 1 — Potato StACS protein sequences. [file DataSheet7.pdf]

Potato ACS Protein sequences:

>Soltu.DM.05G019640.1|Protein

MNGFFLLVAKLQLCYQLLLLLLPNFVRRNLLAIREVFVITSNVVLSELATNEQHGENSLY

FEGWKAYNKDPFDLKDNPNGIIQLGLAENQLSVSMIEDWNKRNLQAFIFSITNQDHGLFE

RLINGIAKLMEKTRGGNVKFDAERIVLTAGATAANETLISCLADPEDAFLIPAPYYTGFD

RDLCWRTGVQLVPISCNNFKITIEAVKEAYEKAQKANIKLKGLILTNPSNPLGTVLDRDT

LKNILTFTNDLVCDEVYAGTVFDAPRFVSIAEIIKENEIYVNKNLVHIVSSLSKDMGLPG

FRAGILYSFNDDVVNFARKRSNIGDLNETQHFLASMLADDEFVQEFLTESEPTLNAEVSL

WKLIINNAKLNISPGSSFHCPEAGWFRICFANIDDQTVEIALQRIRLFVDANVMSSSPN

>Soltu.DM.12G025700.1|Protein

MAIVELSKVANSDTHGEDSPYFAGWKAYDEDPFDEVHNPCGVIQMGLAENQVSFDMVEEY

LEKHSKTINCGSGISNFRENALFQDYHGLYSFRKSMAKFMEKIRGGRAKFNPDRIVITAG

ATAANELLTFILADPGDALLVPTPYYPGFDRDLRWRTGVKIIPIHCNSSNNFQVTPQALE

SAYEEAKVNKIKVRGILITNPSNPLGATIQRSNLEGILDFVVRKNIHLISDEIYSGSAFC

SSEFVSIAEVLESRDDVDSERVHIVYSLSKDLGLPGFRVGTIYSYNDNVVTTARRMSSFT

LISSQTQQLLASMLSNEEFTTNYIKTNRDRLRKRYEKIIDGLKSSGIECLKGNAGKNRRF

HPLSERTFLGLMASKIQQLQSKACQASQFLSKHGTTYYKQLLEQNKQHIVEPPTVEKCNE

LSKQLLYTRLASIPGRYESFWKEVDSVKHLWRNRKELKVEDAGIAALFGLECFAWYCAGE

IVGRGFTFTGYYV

>Soltu.DM.04G032120.1|Protein

MLKMLSKMAMCKSHGQDSSYFIGWQEYEKNPYDPLQNPSGIIQMGLAENQLSFDLLESWL

ARNQDVIQFRENGGSMFRDLALFQDYHGLQAFKNVLVSFMAEIRRKKVKFDPKNLVLTAG

STSANEILIFCLAEPGEALLIPTPYYPGFDRDLKWRTGAEIVPIHCYSSNNFRITESALE

EAYEQAQKRNLTVKAVFITNPSNPLGTTMSRNELNILITFAMTKNIHIVSDEIYAGTVFD

SPKFISIMEALIDRKLEKSKMWNQVHIVSSLSKDLGLPGFRVGMIYSNNETLIAAATKMS

SFGLISSQTQYLLSKILGDRRFIKRYIKENKKQLIHRREMLASGLANSGIECLESNAGLF

CFVDMRDLLKSNTFEAEMELWKKIISNVGLNVSPGSSCHCSEPGWFRVCFANMSKETLDL

AMQRINNFVNSDRDIRQNLRQQPPRSVRAAGSRRRRTIANWVVKLSSGDGRTDR

>Soltu.DM.05G019670.1|Protein

MGSFMSKLQPIKVSSEITSNVVLSKLATNEQHGENSLYFDGWKAYDKDPFHPVNNPNGVI

QLGLAENQLSIDRIEDWIKRNPKASICTHEGIECFRRITNFQDYHGLPEFTTGIAKHMEK

TRHGKVNFDSKRIVMAGGATGANEALIFCLANRGDAFLVPTPYYPGFNRDLCWRTGVQLV

PISCNSSNNFKITPEALKEAYEKGQQEKIKIKGLILTNPSNPLGTTLDRDTLKKILIFTN
EHNIHLVCDEIYAGTVFDAPRFVSIAEIIEDDGICVNKNLIHIVSSLSKDMGFPGFRVGI
VYSFNDDVVNNARKMSSFGLVSTQTQNFLASMLSDDEFIEEFLTENTKRLRKRHEKFTSG
LEKIGIRCLKSNAGVYCWVDLRTLLKEPTLDAEVSLWKLIINNAKLNISPGSSFNCPEAG
WFRICFANIDDRTVEIALKRIQIFVDANNDINENGIVKNASVKGK
>Soltu.DM.02G027270.1|Protein
MKLLSEKATCNSHGQDSSYFLGWQEYEKNPYDEIHNPKGIIQMGLAENQLSFDLLESWLA
QNPDAAGFKRNGESIFRELALFQDYHGLPAFKNAMTKFMSEIRGNKVSFDSNKLVLTAGA
TSANETLMFCLANQGDAFLLPTPYYPGFDRDLKWRTGAEIVPIHCSSSNGFRITESALEE
AYLDAKKRNLKVKGVLVTNPSNPLGTTLTRNELELLLTFIDEKGIHLISDEIYSGTVFNS
PGFVSVMEVLIEKNYMKTEVWERVHIVYSLSKDLGLPGFRIGAIYSNDEMVVSAATKMSS
FGLVSSQTQYLLSCMLSDKKFTKKYISENQKRLKKRHAMLVKGLKNAGINCLESNAGLFC
WVDMRHLLSSNNFDAEMDLWKKIVYDVGLNISPGSSCHCTEPGWFRACFANMSEDTLNLA
MRRMKDFVESTAPNAINHQNQQQSNANSKKKSFSKWVFRLSFNDRQRER
>Soltu.DM.02G007450.1|Protein
MKLLSKKAMCNSHGQDSSYFLGWEEYEKNPYDEIRNPKGIIQMGLAENQLSFDLLESWLT
QNPDAAAFKRNGNSIFRELALFQDYHGLPAFKDALVQFMSEIRGNKVSFDSNKLVLTAGA
TSANETLMFCLADPGDAFLLPTPYYPGFDRDLKWRTGAEIVPIQCTSSNGFRITESALEE
AYKEAERRNLRVKGVLVTNPSNPLGTTLTKKELQLLLTFVSTKQIHLISDEIYSGTVFNS
PKFVSVMEVLIENNYMYTEVWDRVHIVYSLSKDLGLPGFRVGAIYSNDDMVVSAATKMSS
FGLISSQTQYLLSALLSDKKFTKNYVSENQKRLKKRHEMLVGGLKQIGIRCLESNAGLFC
WVDMRHLLSSNTFDGEMELWKKIVYEVGLNISPGSSCHCTEPGWFRACFANMSEDTLNIA
IQRLKAFVDSRDNKDDVQNQKHSNKKKSFSKWVFRLSFNERQRER
>Soltu.DM.07G010590.1|Protein
MAIEIEQRPTVVRLSNVATSDTHGEDSPYFAGWKAYDENPFDEVHNPSGVIQMGLAENQV
SFDLLEEYLEKKKDDGVAEISRFRENALFQDYHGLVCFRKAMATFMEQVRGGRARFDPDT
VVITAGATAANELLTFILADPGDALLVPTPYYPGFDRDLRWRTGVKIIPVHCDSSNNFQV
TLQALEEAYKDAESNNIKVRGVLITNPSNPLGTTVQRCVLEEILEFVARKNIHLVSDEIY
SGSAFCCSEFVSIAEILESRNYKDSERVHIVYSLSKDLGLPGFRVGTIYSYNDKVVTTAR
RMSSFTLISSQTQQLLASMLSDEKFTENYIKKNRERLRRRYEMMIEGLRSAGIECLRGNA
GLFCWMNLSTLLEKPTKECELEVWNTILHEVKLNISPGSSCHCSEPGWFRVCFANMSENT
LEIALKRIHHFMETRGILQKY

>Soltu.DM.01G034180.1|Protein

MGFEIAKTNSILSKLATSEEHGENSPYFDGWKAYDSDPFHPLKNPNGVIQMGLAENQLCL

DLIEDWIKRNPKASICSNEGIKSFKAIANFQDYHGLPEFRRAIAKFMEKTRGGRVRFDPE

RVVMAGGATGANETIIFCLADPGDAFLVPSPYYPAFNRDLRWRTGVQLLPIHCESSNNFK

ITSKAVKEAYENAQKSNIKVRGLILTNPSNPLGTTLDKDTLKSLLSFTNQHNIHLVCDEI

YAATVFDTPQFVSIAEILDEKEMTYCNKDLVHIVYSLSKDMGLPGFRIGIVYSFNDDVVN

CARKMSSFGLVSTQTQYFLAAMLSDEKFVDNFLTESAIRLAKRHKHFTNGLEEVGIKCLK

NNAGLFCWMDLRPLLRESTFDSEMSLWRVIINDVKLNVSPGSSFECQEPGWFRVCFANMD

DGTVDIALARIRRFVRVEKSGDESSAMEKKQQWKKNNLRLSFSKRMYDESVLSPLSSPIP

ASPLVR

>Soltu.DM.03G005280.1|Protein

MATCESHGQNSSYFLGWQEYEKNPYDEIQNPKGIIQMGLAENQLSFDLLESWLAQNPDAA

GFKRNGESIFRELALFQDYHGLPDFKNALVQFMSEIRGNKVTFNPNKLVLTAGATSANET

LMFCLANPGDAFLLPTPYYPGFDRDLKWRTGAEIVPIQCTSSNGFRITQSALEESYKLAK

TRNLRVKGVLVTNPSNPLGTALTRNELELLVSFVAEKGIHLISDEIYSGTVFNSPKFVSV

MEVLIENNYMYTEVWDRVHIVYSLSKDLGLPGFRIGAIYTNDEVIVSAATKMSSFGLISS

QTQYLLSAMLADKKFTKKYISENQKRLKKRHAMLVKGLESTGISCLESNAGLFCWVDMRH

LLKTNTFEAEIELWKKIVYEVKLNISPGSSCHCTEPGWFRACFANMSEDTLNLAIQRIKI

FVDSSDVIGTNIDQSNQTNQNTSTSLKKKLFAKWGFRLSFNDRER

>Soltu.DM.02G007440.1|Protein

MKLLSKKAMCNSHGQDSSYFLGWEEYEKNPYDETRNPKGIIQMGLAENQLSFDLLESWLT

QNPDAAAFKRNGNSIFRELALFQDYHGLPAFKDALVQFMSEIRGNKVSFDSNKLVLTAGA

TSANETLIFCLADPGDAFLLPTPYYPGFDRDLKWRTGAEIVPIQCTSSNGFRITESALEE

AYKEAERRNLRVKGVLVTNPSNPLGTTLTKKELQLLLTFVSTKQIHLISDEIYSGTVFNS

PKFVSVMEVLIENNYMYTEVWDRVHIVYSLSKDLGLPGFRVGAIYSNDVMVVSAATKMSS

FGLISSQTQYLLSALLSDKKFTKKYVSENQKRLKKRHEMLVGGLKQIGIRCLESNAGLFC

WVDMRHLLSSNTFDGEMELWKKIVYEVGINISPGSSCHCTEPGWFRACFANMSEDTLNIA

IQRLKAFVDSRDIQNQQHSNKKKSFSKWVFRLSFNERQRER

>Soltu.DM.08G028300.1|Protein

MVSISNNNQKQQLLSKIATNDGHGENSPYFDGWKAYANDPFHLTDNPNGVIQMGLAENQL

CFDLIQEWVVNNPKASICTVEGAENFQDIAIFQDYHGLPEFRQAVAKFMEKVRGDKVTFD

PDRIVMSGGATGAHEMLAFCLADPGDAFLVPTPYYPGFDRDLRWRTGVQLFPVVCESCND

FKVTKKALEEAYEKAQQSNIKIKGLLINNPSNPLGTLLDKDTLQDIVTFINSKNIHLVCD

EIYAATVFDQPRFISVSEIVEEMIECNKDLIHIVYSLSKDLGFPGFRVGIVYSYNDTVVN

IARKMSSFGLVSTQTQHLLASMLSDEVFIEKFIAESSERLGERQGMFTKGLAEVGISTLK

SNAGLFFWMDLRRLLKEATFDGELELWRMIINEVKLNVSPGCSFHCSEPGWFRVCFANMD

DETMRIALKRISYFVLQPKGLNNKAAVNKQCSRRKLQISLSFRRLDHEFMNSPAHSPMNS

PLVRT

>Soltu.DM.12G008180.1|Protein

MGFISNINNELLSKVATNNGHGENSAYFDGWKAYEIDPFHPTQNPNGVIQMGLAENQLCF

DLIQEWVVNNQKSSICTAGGCEEFKEIAIYQDYHGLPEFRRAVARFMEKVRGDKIKFDEE

RIVMSGGATGAHELLAFCLADPGEAFLVPTPYYPG

>Soltu.DM.03G016130.1|Protein

MRLVVPLQGVVQGRGGLILGSLIPCALFYFLQFYSKRHRTTPSSSNPPSPSTSSPNLSEL

QRSSSRLNLSTRGSVGRVFLSSRASLVAAPNDSPYYIGMDRFRADPYDELDNPDGVIDLG

IAENRLSLDLIEKWISSNVNGSTLGSCGDGLNINGILTYQPFDGLAELKVAMAGFMSQVM

GEKVSFDPSRMVLTSGATPAIEVLCFCLADHGNALLVPTPYYPGFDRDIRWRTGVDLIPV

HCRSSDAFMVDIIALDQAFSHARKRGQKVRGILISNPSNPVGNIMSRETLCRILDFAREK

NIHVISDEIFAGSNYGGTEFVSIAEILDEEDPDRDRVHIIYGLSKDLSVPGFRLGVLYSF

NENVVAASKKLTRFCAASAPTQSLLVAMLSDAGFIKDYMRTNRERLRKVFDLFVAGLKQL

GIECMNSSAGLYCWVNMSGLICPYNEKGELELWEKLLNVAKINVTPGSACHCIEPGWFRC

CFSTVEEKDIPVVMERIRKVVERS

>Soltu.DM.08G004500.1|Protein

MGLISENYNKNLLSKIATNDGHGENSAYFDGWKAYENDPFHPTQNPHGVIQMGLAENQLC

FDLIQEWIVNNPKASICTYEGVQDFQDIAIFQDYHGLPEFRKAVARFMEKVRGDRVTFDP

ERIVMSGGATGAHESLAFCLADPGDAFLVPTPYYPGFDRDLRWRTGVQLFPVVCESYNNF

KVTKEALEEAYKKAQESNIKVKGLLINNPSNPLGTILDKETLKDILRFINDKNIHLVCDE

IYAATAFSQPSFISISEVMNEVVGCNDDLVHIVYSLSKDLGFPGFRVGIIYSYNDVVVNI

ARKMSSFGLVSTQTQRLIASMLSDTIFVENFIAKSAMRLSQRHDLFTIGLGQVGITTLKS

NAGLFIWMDLRRFLEKSTFDDELKLWHIIINKVKLNVSPGCSFHCSEPGWFRVCFANMDD

ATMQVALKRIRHFVYLQPSKGVEVATKKQYCRTRSKLEISLSFRRLDDFMNSPHSPMSSP

MVQARN

>Soltu.DM.08G026280.1|Protein

MTRSRDRSPTRTTTTTISTGGAGGRDGGGATTAMRVIVPLQGVVQGRGGLFLGSVIPCAL

FYFWQLYLKRNRSSGGDNNGESTAPARSTSSTHLPEVSSGSGLQRVHSRVLLSPKGTTGQ

SQVSARANSIISKQIDSSPYYVGLKRASEDPYDESSNPDGVIQLGLAENKLSLDLVQEWL

AENVSRWMMTQDSSITGIATYQPFDGLLELKVAVGEFMSQALERSVSFSPSQMVLTGGAA

PALEILSFCLADPGNAFLVPSPYYPDLDRDVKWRTGVEIIPVPCRSADNFNLSIDALDRA

FNQAKKRGLKVRGIIISNPSNPVGNIFSRETLYNLLDFATEKNIHVISNEILAGSTYGRE

EFVSIAEIIDSEDFDRSRVHIVYGLSKDLSLPGFRVGVIYSCNENVLAAAKKLTRFSSIS

APTQHLIIQMLSDAKFVQQFIKKNRERLRKMSSLFVSGLKQLGIECTKSSGGFYCWADMS

RLIRSYNEKGEIELWDNLLNVAKINATPGSSCHCVEPGWFRLCFSTLSEKDISTVMQRIQ

KVLELRKSLS

>Soltu.DM.08G026280.2|Protein

MTRSRDRSPTRTTTTTISTGGAGGRDGGGATTAMRVIVPLQGVVQGRGGLFLGSVIPCAL

FYFWQLYLKRNRSSGGDNNGESTAPARSTSSTHLPEVSSGSGLQRVHSRVLLSPKGTTGQ

SQVSARANSIISKQIDSSPYYVGLKRASEDPYDESSNPDGVIQLGLAENKLSLDLVQEWL

AENVSRWMMTQDSSITGIATYQPFDGLLELKVAVGEFMSQALERSVSFSPSQMVLTGGAA

PALEILSFCLADPGNAFLVPSPYYPEDVKWRTGVEIIPVPCRSADNFNLSIDALDRAFNQ

AKKRGLKVRGIIISNPSNPVGNIFSRETLYNLLDFATEKNIHVISNEILAGSTYGREEFV

SIAEIIDSEDFDRSRVHIVYGLSKDLSLPGFRVGVIYSCNENVLAAAKKLTRFSSISAPT

QHLIIQMLSDAKFVQQFIKKNRERLRKMSSLFVSGLKQLGIECTKSSGGFYCWADMSRLI

RSYNEKGEIELWDNLLNVAKINATPGSSCHCVEPGWFRLCFSTLSEKDISTVMQRIQKVL

ELRKSLS

>Soltu.DM.08G028290.1|Protein

MVSISNNNNQKQQLLSKIATNDGHGENSPYFDGWKAYANDPFHLTDNPNGVIQMGLAENQ

LCFDLIQEWVVNNPKASICTAEGAENFQDIAIFQDYHGLPEFRQAVARFMEKVRGDRVTF

DPDRIVMSGGATGAHEMLAFCLADPGDAFLVPTPYYPGFDRDLRWRTGVQLFPVVCESCN

DFKVTKKALEEAYEKAQQSNIKIKGLLINNPSNPLGTLLDKDTLRDIVTFINSKNIHLVC

DEIYAATVFDQPRFISVSEIVEEMIECNKDLIHIVYSLSKDLGFPGFRVGIVYSYNDTVV

NIARKMSSFGLVSTQTQHLLASMLSDEIFIERFIAESSERLGQRQGMFTKGLAQVGISTL

KSNAGLFFWMDLRRLLKEATFDGELELWRIIINEVKLNVSPGCSFHCSEPGWFRVCFANM

DDETMKTALRRIRNFVLQTKGLNNIAAIKKQCSRSKLQISLSFRRLDDFNSPAHSPMNSP

LVRT
