# Supplementary material for: StACS3-mediated drought stress adaptation in potato involves interactions with StPP2C2 and St14-3-3 proteins
Source: Front Plant Sci. 2025 Oct 30;16:1671817. doi: 10.3389/fpls.2025.1671817 (PMC12611960; doi:10.3389/fpls.2025.1671817)
Supplement: Supplementary File 2 — Supplementary figures legends. [file DataSheet8.pdf]

Supplementary Figure 1. Relative gene expression and promoter activity analysis of StACS3 using GUS reporter assays.

(A) Five- to six-week-old potato plants were subjected to water stress for 14 days. Drought-resistant and drought-sensitive cultivars were analyzed under these conditions (Schumacher et al., 2021). Transcript accumulation of StACS3 and other candidate genes was assessed by semi-quantitative RT-PCR, with Potato Ubq used as an internal control. Densitometric values below the bands represent expression levels normalized to Ubq.

(B) *Nicotiana benthamiana* leaves were infiltrated with pStACS3::GUS via *Agrobacterium tumefaciens* and harvested 2–3 days post-infiltration (dpi). The leaves were then treated with PEG for 4–5 hours to simulate osmotic stress. PEG treatment induced GUS expression (right panels), whereas no expression was observed in the untreated samples (left panel). The Agrobacterium OD600 was 1.5 in the top right panel and 1.0 in the lower right and mock-treated panels.

(C) GUS activity in infiltrated leaves under PEG-induced stress was visualized using transmitted light confocal microscopy (20x magnification), and images were processed using ImageJ.

(D) GUS expression driven by the StACS3 promoter in transgenic *Arabidopsis thaliana* (line no. 4) was analyzed following exposure to 4°C (cold stress) and 37°C (heat stress).

Supplementary Figure 2. Localization and tissue-specific expression of StACS3-YFP in *Arabidopsis thaliana* transgenic plants.

Confocal microscopy reveals StACS3-YFP localization in sexual organs (a), stomata, and punctate structures, with mild expression at the plasma membrane in leaf cells (b), and predominant localization in the stomata of floral organs (c, d).
